# Atomic-Level Investigation of Reactant Recognition Mechanism and Thermodynamic Property in Glucosamine 6-Phosphate Deaminase Catalysis

**DOI:** 10.3389/fchem.2021.737492

**Published:** 2021-08-03

**Authors:** Xiao Zhang, Xiaoyuan Liu, Zhiyang Zhang, Yuan Zhao, Chaojie Wang

**Affiliations:** The Key Laboratory of Natural Medicine and Immuno-Engineering, Henan University, Kaifeng, China

**Keywords:** glucosamine 6-phosphate deaminase, reactant recognition and delivery, lid motif, umbrella sampling, hydrogen bond dependent

## Abstract

Glucosamine 6-phosphate deaminase (NagB) influences the direction of N-acetylglucosamine (GlcNAc) metabolism, facilitating the conversion of D-glucosamine 6-phosphate (GlcN6P) to D-fructose 6-phosphate (Fru6P) with the release of ammonia. Here, extensive molecular dynamics simulations combined with various techniques were performed to study the recognition and delivery process of GlcN6P by *Smu*NagB, due to its guidance of subsequent enzymatic reaction. The key residues Lys194, His130, Arg127, Thr38, and Ser37 stabilize GlcN6P in the active site by hydrogen bond interactions, therein electrostatic and polar solvent effects provide the primary traction. Four delivery channels were identified, with GlcN6P most likely to enter the active site of NagB through a “door” comprising residues 6–10, 122–136, and 222–233. The corresponding mechanism and thermodynamic properties were investigated. An exothermic recognition and delivery process were detected, accompanied by the flipping of GlcN6P and changes in key direct and indirect hydrogen bond interactions, which provide the driving force for the chemical reaction to occur. Furthermore, “the lid motif” was identified that remain open in alkaline condition with different extent of opening at each stage of transfer that induced GlcN6P to move the active site of NagB. The work will assist in the elucidation of the catalytic mechanism of action of NagB, allowing inhibitors to be designed with superior dynamic behavior.

## Introduction

**FIGURE 1:**
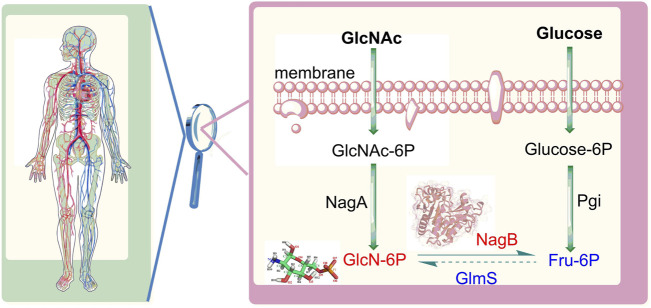
Distribution of sugars mediated by glucosamine-6-phosphate deaminase (NagB) and glutamine-fructose-6-phosphate aminotransferase (GlmS). GlcNAc: N-acetylglucosamine, GIcNAc-6P: N-acetylglucosamine 6-phosphate; GlcN-6P: glucosamine-6-phosphate; Fru-6P: fructose 6-phosphate; Glucose-6P: glucose 6-phosphate; NagA: N-acetylglucosamine 6-phosphate deacetylase Pgi: glucose 6-phosphate isomerase.

Aminosugars are abundantly present in nature and function as supplier of carbon and nitrogen during bacterial growth and proliferation. The galactosamine/acetylgalactosamine (GalN/GalNAc) catabolic pathway is a vital component of aminosugar metabolism, in which glucosamine-6-phosphate deaminase (NagB) functions as the primary metabolic enzyme. A catabolic enzyme in most organisms, NagB carries out an isomerization and deamination reactions, facilitating the conversion of D-glucosamine 6-phosphate (GlcN6P) to D-fructose 6-phosphate (Fru6P) with the release of ammonia (Assrir et al., 2014; Yoo et al., 2016; Zhang et al., 2015) (Figure 1). The final step is the utilization of N-acetylglucosamine which defines metabolic direction, affecting the synthesis of lipopolysaccharides and the recycling of amino sugars on the cell wall, in addition to the production of bacterial cell membranes.

GlcN6P deaminase is expressed in many different organisms, while it is absent from several green plants and eukaryotic lineages (Burki et al., 2020). The enzyme was originally described in extracts of pig kidney (Yu et al., 2020) then subsequently in a number of other organisms, including *Escherichia coli* (Jang et al., 2017), *Candida albicans* (Arkowitz and Bassilana, 2019), *Musca domestica* (de Jonge et al., 2020), Plasmodium falciparum (Neveu et al., 2020), *Canis familiaris* (Guedes et al., 2021), Bos Taurus (Hu et al., 2020), and *Homo sapiens* (Weni 2020). Over the years, the crystal structure of the NagB hexamer was elucidated in *Escherichia coli* and *Homo sapiens*, while the crystal structure of the NagB monomer was obtained from *Bacillus subtilis* and *Streptococcus mutans* (Liu et al., 2008). NagB from *Streptococcus mutans* (*S. mutans*) is encoded by the *smu*.636 gene, and has 41.7% homology with *Bsu*NagB (Liu et al., 2008). The Gram-positive bacterium *S. mutans* is the principal pathogen in human dental caries (Ito et al., 2019) and relevant to nonoral infections such as subacute bacterial endocarditis (Schmalzle 2020). Enzymes participating in peptidoglycan biosynthesis are considered important and have become a focus of research on the identification of antibacterial agents (Strassert et al., 2019). These insights provide evidence for the catalytic mechanism of NagB, and contribute to the elucidation a more comprehensive understanding of the mechanisms of action of NagB, providing criteria for the design of antimicrobial agents for the dental pathogen *S. mutans*.

In previous studies, an enzymatic ring-opening mechanism was identified in GlcN6P in a basic environment (pH = 8.0), for which *Smu*NagB exhibited the greatest catalytic activity when analyzed by ab initio quantum mechanics/molecular mechanics (QM/MM) molecular dynamics (MD) simulations. In addition, we established that the effect of the pH and found “lid motif” probably exhibited varying flexibility with substrate binding that varied at different pH values using four classical molecular dynamic trajectories of the apo state and complex state in acidic and basic solution (Zhao et al., 2014). The current study provides the basis for elucidating the mechanisms of the chemical reactions of NagB and explores initial speculation about the performance of the “lid motif”. In some enzymatic systems, non-bonded interactions involved in reactant (or product) delivery may determine catalytic efficiency or provide the key driving force for enzymatic catalysis. Here, we principally focused on the dynamic reactant (GlcN6P) transport process essential for enzymatic catalysis to occur. Additionally, a number of crucial open issues have attracted our attention, as follows. The “lid motif” in NagB is important for substrate binding and most likely experiences typical conformational changes in experimental predictive modeling. Our MM MD calculations comparing of the apo state and complex state predicted that, according to the protonated state of key residues in different environments, the lid motif mostly maintains an “open” state in basic solution; while moving from the “open” to the “closed” state upon substrate binding in acidic solution. That method did not involve the entire dynamic substrate transport simulation only providing an initial prediction, and thus important unknowns, including whether the performance of the “lid motif” in environments with the highest catalytic efficiency, in addition to the ingress of the substrate from outside to the active site of NagB, results in only a small change or a significant difference in a particular conformation. In addition, it is unknown whether the initial recognition of substrate in the cavity includes the “lid motif”. If it does, the cavity comprises five other fragment that refer to residues 6–10, 122–136, 150–163, 189–198, and 222–233. Thus, which fragment is more favorable for binding the substrate? If the cavity is not inclusive, which channel is most favorable? Moreover, differences in performance of the “lid motif” in different environments was found in a previous study to be associated with different states of protonation of His145, whereas key residues will possibly change in response to differences in the predominant substrate and mode of delivery, in which case which factors influence the degree of difficulty of substrate binding? Furthermore, it should also be noted that the ring-opening of GlcN6P requires a barrier of approximately 18.0 kcal/mol to overcome, so where does the driving force for this reaction originate? It remains to be determined whether substrate transportation can provide sufficient energy, and how about the thermodynamic and dynamic properties of the process? What factor represents the key source for changes in free energy? Solving these questions from a microscopic viewpoint, will be particularly meaningful for understanding the entire enzymatic catalytic of NagB and the GlcN6P metabolism, and will provide a critical molecular basis for designing drugs targeting *Smu*NagB.

## Computational Methods

### Construction of Complex Model

**FIGURE 2:**
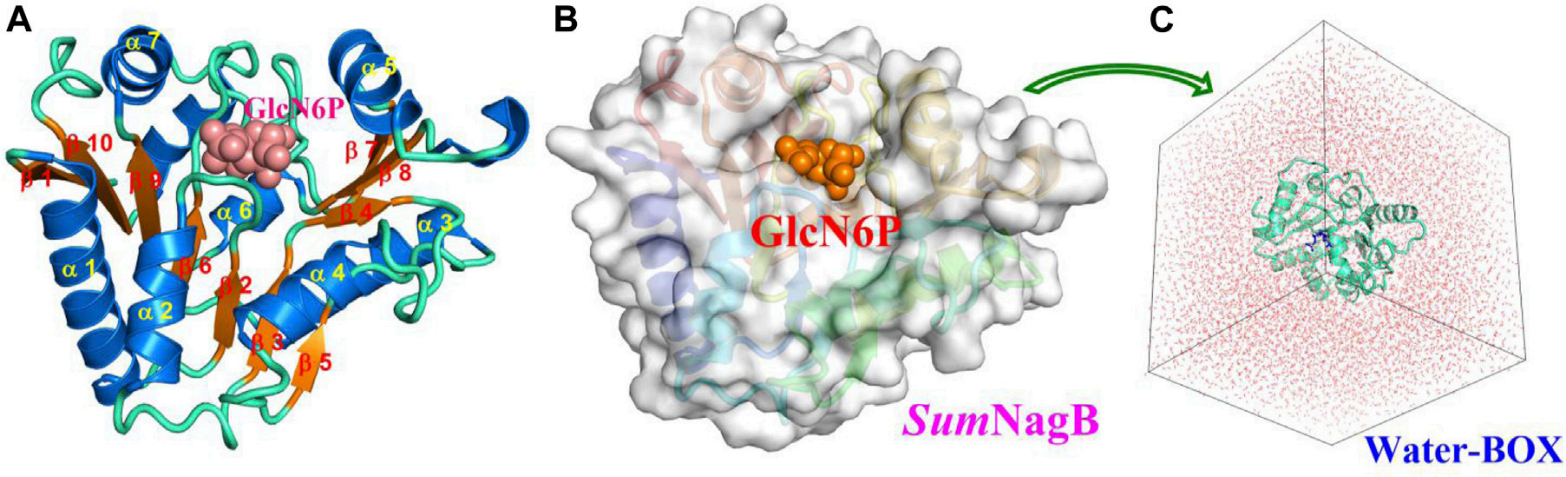
Crystal structure **(A)** and the corresponding surface model **(B)** of *Smu*NagB with GlcN6P in addition to the initial model from MM MD simulations **(C)**. Helices are blue and numbered α1 to α7; β-strands are orange and numbered β1 to β10. GlcN6P is shown as a cube.

The initial model was built based on the *Smu*NagB-GlcN6P complex in a basic solution (pH = 8.0) for which the enzyme exhibits greatest catalytic efficiency, and in which protonated of charged residues is the same as that reported in a previous study (Zhao et al., 2014). The GlcN6P is described by the AMBER GAFF force field (Wang et al., 2004; Darian and Gannett, 2005), while *Smu*NagB is treated with the AMEBR99SB force field (Hornak et al., 2006; Wang et al., 2000). The same force field we chose as the previous study (Zhao et al., 2014) may be more comparable. Partial atomic charges of the ligand were computed using Gaussian 09 software (Frisch et al., 2009) using restrained electrostatic potential (RESP) (Jia and Li, 2019) charge (Cornell et al., 1993) at the HF/6–31G(d) level. The entire system is solvated into a 73 × 75 × 74 Å cube of water using the (Ong and Liow, 2019) TIP3P model (Jorgensen et al., 1983) with a 10 Å buffer of distance on each side, and which was neutralized by the addition of Na^+^ ions (Figure 2). The initial coordinates and topological parameters were generated by adding protons automatically using the tleap module (Case et al., 2005) in AMBER 16 software (Case et al., 2016).

### Molecular Dynamics Simulations

To correct unfavorable inter-atomic interactions, the entire system was minimized using 10,000 steps by employing the steepest method of descent, while another 10,000 steps were conducted *via* the conjugate gradient method. Following two-step optimization, the temperature was gradually heated from 0 to 300 K for 100 ps using an NPT ensemble. An additional 100 ps MD simulation was conducted to relax the system density to approximately 1.0 g/cm3. Finally, a 100 ns MD simulation is executed using an NVT ensemble with an integrating time step of 1 fs based on the periodic boundary conditions. Long-range electrostatic interactions were manipulated using the particle mesh Ewald (PME) method (Linse and Linse, 2014), in which the temperature is maintained at 300 K *via* the Langevin method. A 12 Å cutoff was established to count van der Waals and electrostatic interactions. The SHAKE algorithm (Ryckaert et al., 1977) was applied to constrain all hydrogen-containing bonds within a tolerance of 10^−5^. The stability of the backbone of *Smu*NagB was determined in terms of root mean square deviation (RMSD) while hydrogen bonds were analyzed through the use of equilibrium trajectories from MM MD simulations. All the molecular dynamics simulations were conducted using the AMBER 16 software (Case et al., 2016).

### Molecular Mechanics/Generalized Born Surface Area Calculations

Molecular mechanics/generalized Born surface area (MM/GBSA) methods represent broad scoring schemes for the calculation of binding free energy and have demonstrated favorable performance for ranking binding affinity (Duchêne et al., 2014; Li et al., 2012; Oliveira et al., 2013). In accordance with previous experiments, an amended generalized Born model (GB_OBC1_) (Xia et al., 2002) proposed by Onufriev and his co-worker (Feig et al., 2004; Onufriev et al., 2004) was adopted to account for polar solvation energy. Here, 100 snapshots were extracted from the final 10 ns equilibrium trajectory of the MM MD simulations, computed using the MM/GBSA method for binding free energy decomposition. An interior dielectric constant of 1.0 was selected for the molecule while a solvent dielectric constant of 78.5 was chosen. A maximum distance of 25 Å for atomic pairs involving the Born radius was assumed, while a surface tension of 0.005 kcal/mol/Å^2^ was used in calculating the non-polar contribution to free energy of solvation. For all calculations, AMBER 16 software was utilized (Case et al., 2016).

### Random Acceleration Molecular Dynamics Molecular Dynamics Simulations

Elucidation of ligand binding and release pathways in protein research is fundamental to drug discovery and therapeutic applications (Lüdemann et al., 2000; Vashisth and Abrams, 2008). Random acceleration molecular dynamics (RAMD) simulation is a wide-ranging technology often utilized in such applications. To identify the predominant substrate release channel from the active core, a theoretical study using a RAMD simulation was undertaken using Nanoscale Molecular Dynamics (NAMD) 2.9 software (Phillips et al., 2005). In RAMD MD simulations, a constant force in a random direction is applied to the center of mass of the ligand. If the ligand moves further than the threshold distance, the direction remains constant. Conversely, a new random direction is applied to define the release route of the ligand. By employing RAMD simulations, a feasible escape route will automatically be identified for the ligand. After escape of the ligand from its initial position, the classic MD simulations were initiated to restore equilibrium sampling using random accelerations of 0.50, 0.45, 0.40, 0.35, 0.30, 0.25, and 0.20 kcal/Å/g, with thresholds of 0.2, 0.3, 0.4, and 0.5 Å, respectively. Lastly, 112 RAMD MD trajectories were obtained, and the substrate successfully escaping from protein needs 320–2000 ps on basis of above random acceleration and thresholds.

### Umbrella Sampling

After establishing the optimal channel delivery by RAMD, the mechanism of ligand delivery, and the thermodynamic and kinetic properties, in addition of essential residues were identified by MM MD simulations combined with umbrella sampling. Using the most favorable channel as determined by the RAMD simulation, the distance between the C5 atom of the ligand and the Cα of Ser175 was selected as the reaction coordinate (RC) for substrate transportation, varying from 12.0 Å to 28.0 Å. There was 0.5 Å between adjacent windows. A total of 33 windows were selected. For each window, a 20 ns MM MD simulation was performed in which harmonic bias potential was applied to ensure that the sampling achieved reasonable overlap. The final 10 ns of the reaction coordinate for all windows were analyzed using the weighted histogram analysis method (WHAM) (Shi et al., 2015; Kumar et al., 1992) to generate the potential of mean force (PMF). All calculations were performed by using AMBER16 software (Case et al., 2016).

## Results and Discussion

### *Smu*NagB-D-Glucosamine 6-Phosphate Complex

**FIGURE 3:**
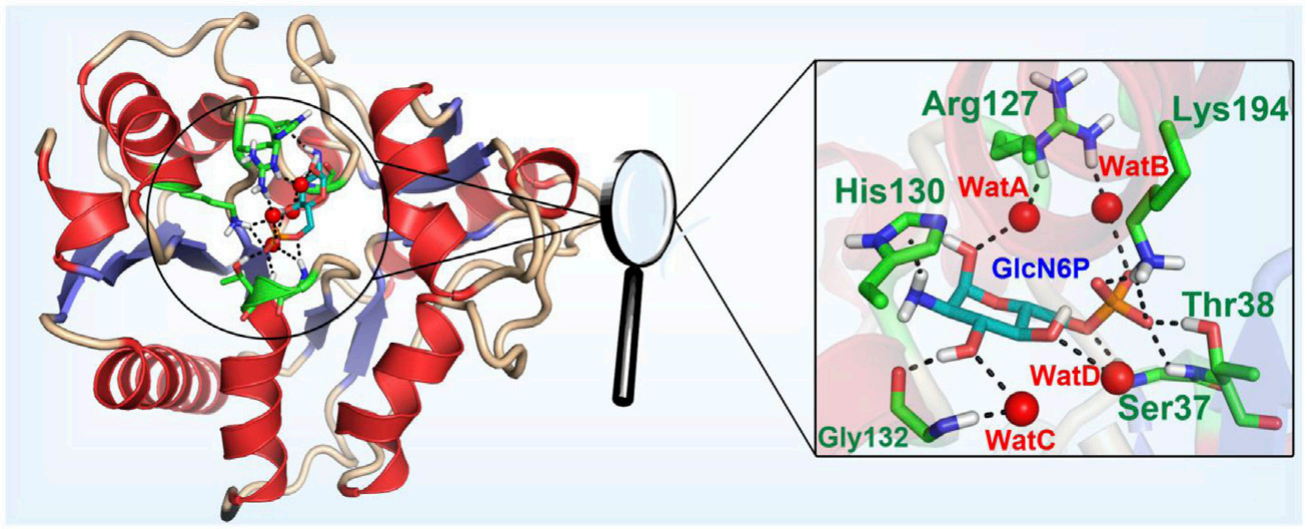
Mode of binding of GlcN6P in the active site of *Smu*NagB.

The model of the *Smu*NagB-GlcN6P complex reached equilibrium after 12 ns during MM MD simulations using root mean square deviation (RMSD) of the backbone atoms, while the final 10 ns of the trajectory was extracted for subsequent analysis (Supplementary Figure S1). A stable snapshot of the MM MD simulations is displayed in Figure 3 and Supplementary Figure S2. GlcN6P is anchored to the active site of *Smu*NagB, where it interacts with six key amino acid residues, forming hydrogen bonds, water-bridged hydrogen bonds, and σ-π bonds. The five-membered imidazole ring of His130 forms a σ-π bond with the small molecular imino group of GlcN6P. The phosphate group of GlcN6P interacts not only with Lys194, Thr38, and Ser37 *via* hydrogen bonds, but also forms a water-bridged hydrogen bond with Arg127 that stabilizes the substrate in the active center.

To obtain more detailed data about the formation of hydrogen bonds between *Smu*NagB and GlcN6P to further ascertain the contribution of the different interactions, the probability of interactions was predicted on the basis of 5,000 snapshot of the final 10 ns of the MM MD simulations. The number of key atoms listed in Table 1 is listed in Supplementary Figure S2. In particular, there was a 93.0% probability of hydrogen bond formation between Lys194 and the phosphate group of GlcN6P. we identified a high probability (83.7%) of hydrogen bond between Thr38 and GlcN6P, which mainly acted to stabilize the phosphate group. With a probability of 73.4, 60.8, and 7.6%, respectively, three hydrogen bonds formed between Gly132 and the hydroxyl group of GlcN6P, stabilizing the substrate at the active site by constraining the six-membered carbohydrate ring. A further three hydrogen bonds formed between Ser37 and different atoms of GlcN6P with a lower probability of 26.1, 22.9, and 12.1%, respectively, focusing on the phosphate group, with a relatively weaker interaction. Subsequently, the rate of hydrogen bond formation between His130 and GlcN6P was only 7.8%, implying that their interaction was weakest and most unstable, and so the contribution of His130 was lower than those of the residues described above. The associated data is displayed in Table 1.

**TABLE 1:** Hydrogen bonds formed between *Smu*NagB-GlcN6P with the corresponding proportion from the final 10 ns trajectory in MD simulations.

Donor	Acceptor H	Acceptor	Proportion (%)
O8@GlcN6P	H@Lys194	N@Lys194	93.0
O6@GlcN6P	H@Thr38	N@Thr38	83.7
O@Gly132	H11@GlcN6P	O2@GlcN6P	73.4
O@Gly132	H11@GlcN6P	N1@GlcN6P	60.8
O7@GlcN6P	H@Ser37	N@Ser37	26.1
O5@GlcN6P	H@Ser37	N@Ser37	22.9
O6@GlcN6P	H@Ser37	N@Ser37	12.1
N@His130	H12@GlcN6P	O3@GlcN6P	7.8
O@Gly132	H9@GlcN6P	N1@GlcN6P	7.6

**FIGURE 4:**
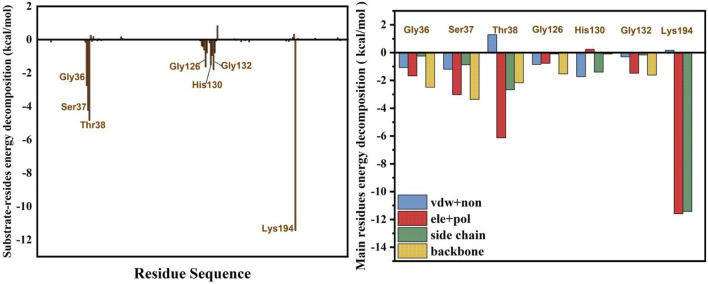
Binding free energy decomposition for the residues.

The binding free energy of the *Smu*NagB-GlcN6P complex was calculated using the MM/GBSA method, based on the binding free energy of decomposition of Gly36, Ser37, Thr38, Gly126, His130, Gly132, and Lys194 dominant residues, as presented in Figure 4. The binding free energy of the *Smu*NagB-GlcN6P complex was −24.4 kcal/mol, due mainly to electrostatic interactions and side chain effects. Both electrostatic and polar effects were strongest for Thr38, presumably associated with hydrogen bond interactions formed with GlcN6P. The next strongest electrostatic and polar interactions of Lys194 were due to the interaction between the Lys194 side chain and GlcN6P. Although the remaining interactions were weaker, they also contribute to stabilization of GlcN6P in the active pocket.

These observations were combined with the effects of virtual alanine scanning mutagenesis of key residues at the active site, as shown in Table 2. Mutation of Lys194 caused a decrease of 23.8 kcal/mol in binding free energy compared with the wild-type enzyme, the greatest decrease of four mutants, indicating that this residue was important. Secondly, more important is Thr38, which caused a decrease in binding free energy of 9.27 kcal/mol. The changes in binding free energy in mutant systems concurred perfectly with hydrogen bonds analysis, which confirmed the significance of the key residues Lys194 and Thr38. As shown in Supplementary Figure S4, when Lys194 was mutated, the interaction with GlcN6P disappeared completely. Mutation of Ser37 and Thr38 resulted in the free energy of key residues also decreasing substantially, in particular Lys194. Consequently, mutation of a key residue also affected the hydrogen bonding network at the binding site. We then performed 100 ns dynamics simulations, respectively, for the four mutated systems (Supplementary Figure S3). Based on the RMSD results of all backbone atoms, each reached a state of the equilibrium. The root mean square fluctuations (RMSFs) of the residues in each system were then calculated. Compared with other mutants, fragments of His130Ala residue displayed the largest fluctuation. It is likely, that a hydrogen bond formed between Arg159 and GlcN6P in the lid motif region, as illustrated in Supplementary Figure S4.

**TABLE 2:** Mean values and standard deviations (M ± SD) of binding free energy of wild type enzyme and four mutant systems (kcal/mol).

System	∆G_gas_	∆G_solv_	∆G_bind_	∆G_wild_ - ∆G_mutant_
WT	−108.11 ± 3.32	83.71 ± 3.21	−24.40 ± 0.83	0
Ser37Ala	−103.21 ± 3.15	79.79 ± 3.05	−23.42 ± 0.83	−0.98 ± 1.30
Thr38Ala	−91.56 ± 3.33	76.43 ± 3.21	−15.13 ± 0.72	−9.27 ± 1.86
His130Ala	−111.15 ± 3.29	90.79 ± 3.21	−20.37 ± 0.74	−4.03 ± 1.45
Lys194Ala	65.14 ± 3.24	−65.75 ± 3.06	−0.61 ± 0.64	−23.79 ± 3.17

### Possible Release Channels of D-Glucosamine 6-Phosphate

It is vital for the entire catalytic process that the substrate enters the active site of the enzyme to facilitate the catalytic reaction. Therefore, the recognition and delivery of GlcN6P by *Smu*NagB, the thermodynamic and kinetic properties, and the interactions between key residues were investigated. More in-depth study of the delivery mechanism will reveal the delivery efficiency of GlcN6P, which is of great importance for the enzymatic reaction process.

**FIGURE 5:**
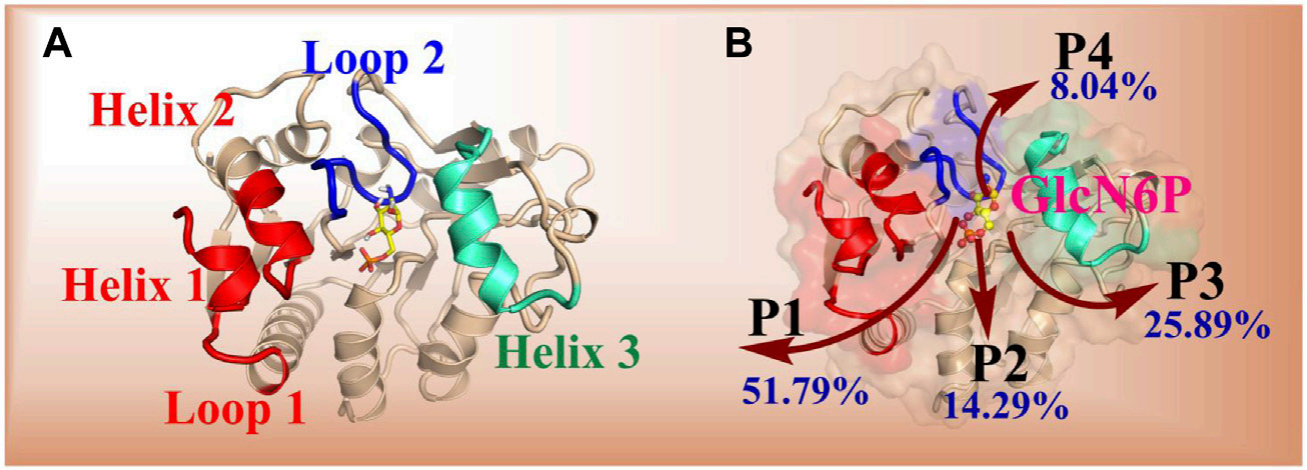
Key segments in the possible channels for the delivery of GlcN6P from *Smu*NagB as determined by RAMD MD simulations. [**(A)**: Loop 1: residues 6–10; Loop 2: 122–136; Helix 1: 222–233; Helix 2: 189–198; Helix 3: 150–163]. **(B)**: red shading indicates the fragments of Loop 1, Helix 1, and Helix 2. Green shading denotes the fragments of Helix 3, and blue shading represents the fragment of Loop 2.

Using RAMD MD simulations, 112 trajectory channels were obtained, divided into four channels according to orientation, namely P1-P4, as displayed in Table 3. Residue segments associated with the GlcN6P channel are displayed in Figure 5 and Supplementary Figure S5. P1, the most favorable channel, accounted for more than half of the probability (51.79%). Located between Loop1, Helix1, and Helix2 it is the most statistically dominant channel. Less favorable are channels P2 and P3, displaying probabilities of 14.29% and 25.89%, respectively. Last is the P4 channel, associated with the Helix3 fragment, with a probability of just 8.04%. Based on these results, the main channel P1 was analyzed more closely.

**TABLE 3:** Location, number, and probability of trajectories for peptide delivery channels from RAMD MD calculations.

Substrate	Location	Share	Probability^a^ (%)
P1	Loop1, helix 1, 2	58	51.79
P2	Helix2, helix3	16	14.29
P3	Helix3	29	25.89
P4	Loop2	9	8.04

a The probability is obtained by the ratio of Nx/Ntotal, where Nx is the number of trajectories of Px, and Ntoal is the number of total trajectories.

### Thermodynamic Characterization and Mechanisms of the P1 Channel

**FIGURE 6:**
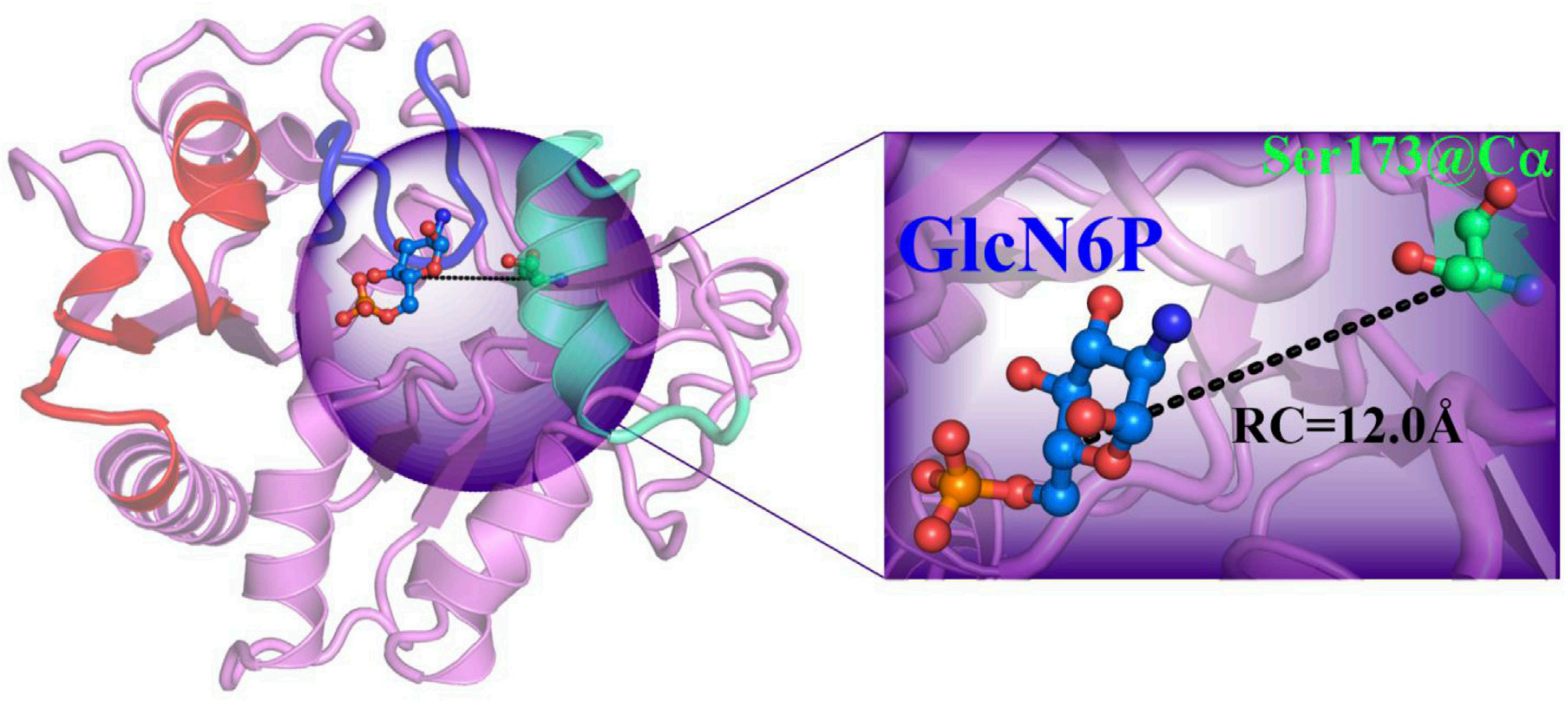
Reaction coordinate definition in umbrella sampling for the most favorable mode of extraction in the P1 direction.

**FIGURE 7:**
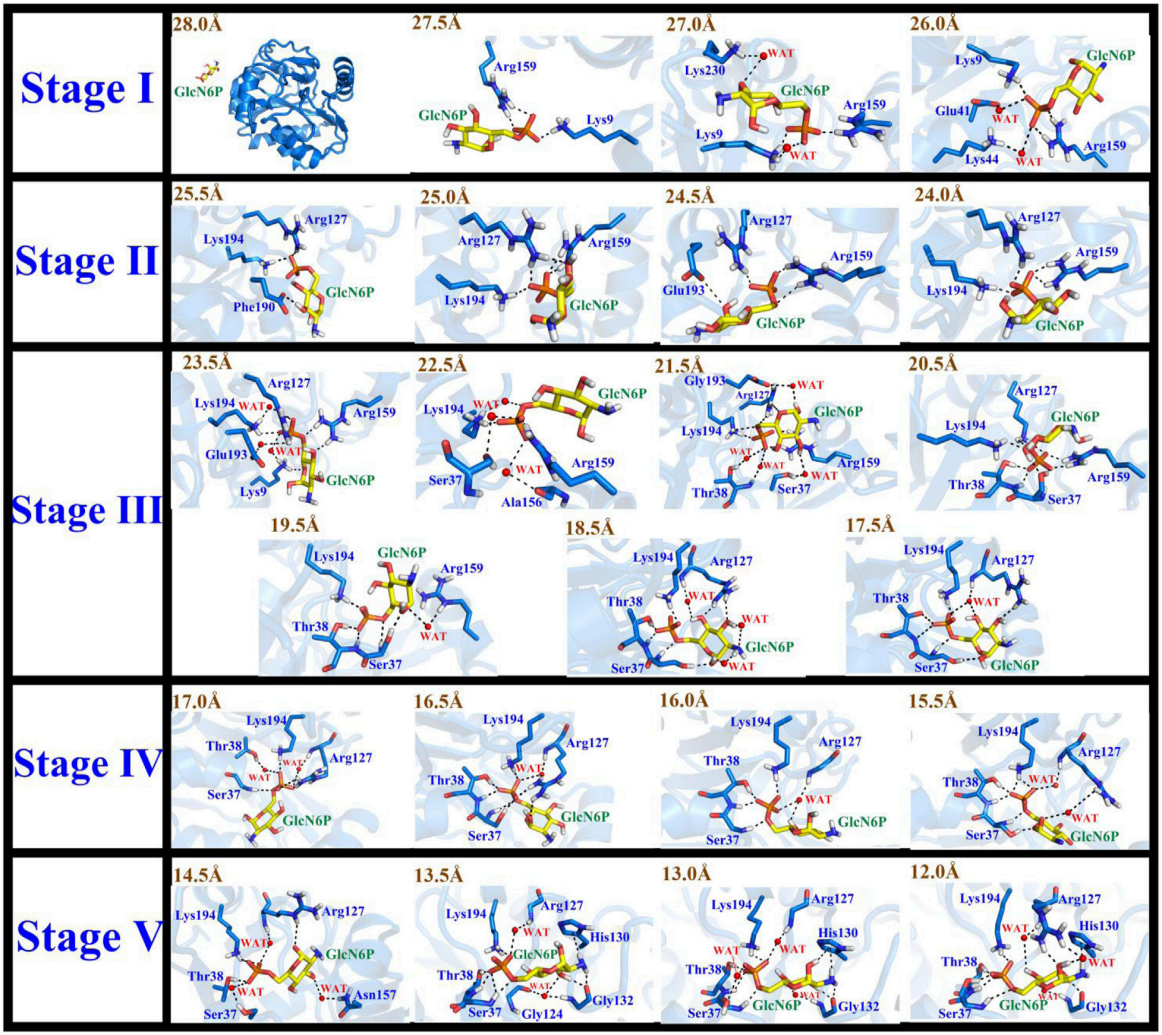
Conformational changes of the substrate and key residues for substrate release along P1 during stages I–V.

**FIGURE 8:**
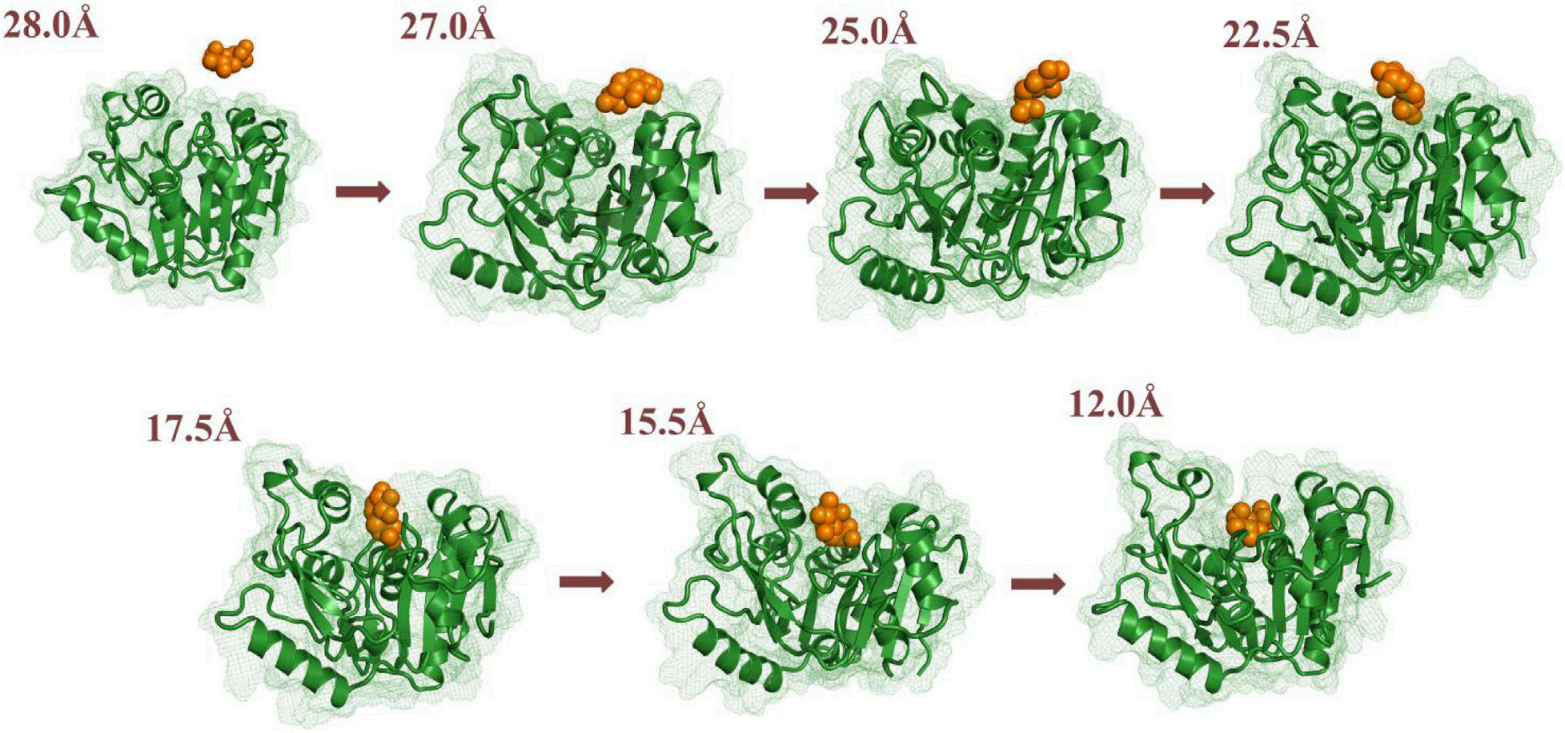
Conformational changes to the substrate and key residues allowing substrate release along P1.

**FIGURE 9:**
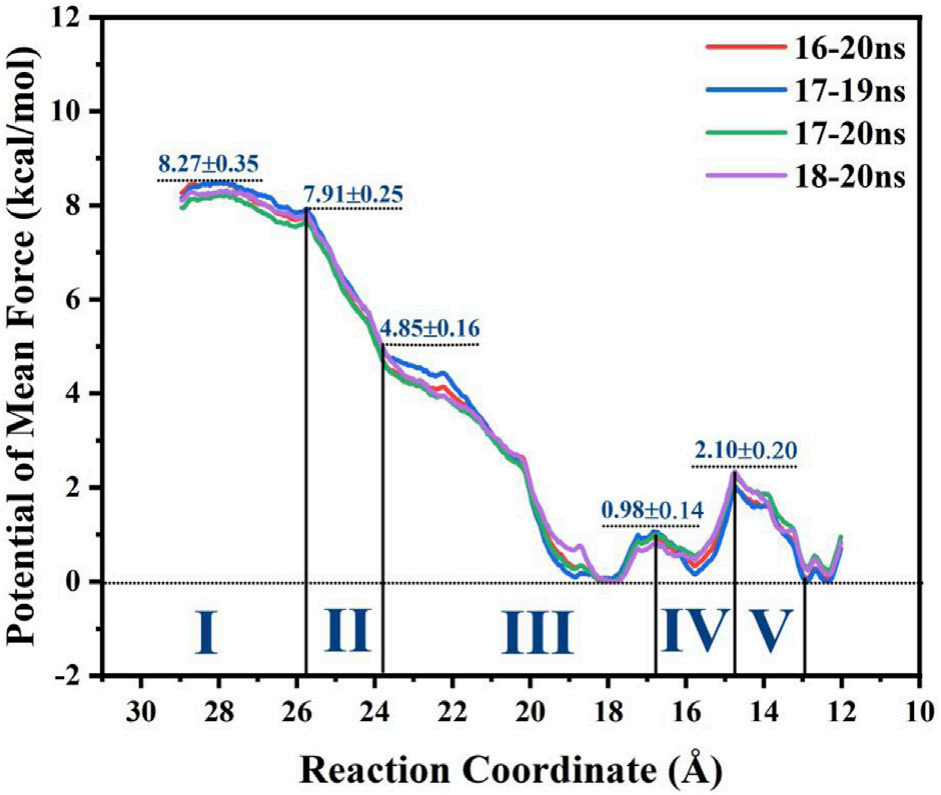
Potential mean force (PMF) of GlcN6P recognition and delivery along the P1 channel evaluated by MM MD simulations incorporating an umbrella sampling technique.

Based on the RAMD simulations, the distance between the Cα atom of Ser173 and the C5 atom of GlcN6P along the P1 channel was defined as the reaction coordinate (RC) (Figure 6). Combining classical MD simulations and umbrella sampling, 33 windows totaling 660 ns at intervals of 0.5 Å were acquired, from 12.0 Å to 28.0 Å. RMSD values indicate that the trajectory was stable (Supplementary Figure S6). A series of harmonic potential type bias tests ensuring that sampling overlapped appropriately (Supplementary Figure S7). Moreover, different time periods are tested to verify the convergence of free energy curves (Supplementary Figure S8). It should be noted that the location of reactant is uncertain, and on basis of our previous study (Zhao et al., 2018), the process of reactant entrance into the active site is similar reversible to the product release in a certain extent, especially for the possible pathways, delivery mechanisms, and conformation changes of protein. Therefore, the analysis of thermodynamic property and mechanism mainly starting from 28.0 Å to 12.0 Å. By analyzing the free energy curve in addition of the key interactions between *Smu*NagB and GlcN6P, as shown in Figures 7–9, the delivery process was divided into five stages.

In the first stage (28.0 Å ≥ RC > 26.0 Å), GlcN6P was unattached and entirely outside *Smu*NagB when RC ≥ 28.0 Å. While entering the cavity, the phosphate groups of GlcN6P preceded the six-membered ring due to hydrogen bond interactions. Lys9 and Arg159 acted as gatekeepers on each side of the entrance, as if holding out their hands to welcome GlcN6P. As new hydrogen bonds form between Lys230 and GlcN6P, the six-membered ring is recognized and flips toward the interior of the cavity. Lys44 and Glu41 also form hydrogen bonds with the phosphate group of GlcN6P. As GlcN6P gradually approaches the pocket, in *Smu*NagB, the number of hydrogen bonds forming increases, consolidating the interaction between GlcN6P and the enzyme, describing the generalized mode by which small molecules are recognized.

In the second stage (26.0 Å ≥ RC > 24.0 Å), as the number of hydrogen bonds between *Smu*NagB and GlcN6P increases, the carbohydrate ring slowly moves toward the active pocket. The conformation of the ring then adopts a horizontal rather than vertical orientation, due mainly to transformation of the hydrogen bonding between Lys194 and the phosphate group, seeking a suitable recognition channel for GlcN6P. Interestingly, Arg127 forms consistent hydrogen bonds with the phosphate group, providing stable traction for GlcN6P. Moreover, both Glu193 and Phe190 help flip the conformation of the hydroxyl group of the hexatomic ring with hydrogen bond interactions. During this stage, direct hydrogen bonding is dominant, while the open lid motif facilitates ingress of GlcN6P into the interior of the cavity.

In the third stage (24.0 Å ≥ RC > 17.0 Å), additional windows were collected, a period critical for identification of the six-membered ring. Formation of additional direct and indirect hydrogen bonds, *via* water molecules, resulted in a clear flipping of GlcN6P, events that define this stage. During recognition, hydrogen bond formation between Arg159 and the substrate becomes gradually weakened, then eventually disappears. It should be noted that Arg159 is located within the “lid motif”, associated with the “door” allowing substrate ingress. The date, therefore, indicate that GlcN6P approaches the inside of the cavity gradually. Hydrogen bonds and water bridging hydrogen bond interactions between Arg127 and GlcN6P influence the flipping of the carbohydrate ring. Meanwhile, the conformational changes in GlcN6P are also associated with Ser37 and Thr38. During this process, the number and strength of hydrogen bonds gradually increase. The interaction between Lys194 and GlcN6P throughout this phase guides GlcN6P to its destination. Newly generated hydrogen bonds provide traction for GlcN6P to gradually approach the interior of the active pocket. Other Residues, including Lys9, Ala156, and Glu193 also contributed to the entry pocket of GlcN6P. This stage is, consequently, of extreme importance in the entire process of recognition process.

In the fourth stage (17.0Å ≥ RC > 15.0 Å), the number of hydrogen bonds decreases compared with the previous stage, mainly due to breakage of hydrogen bonding at the entrance. GlcN6P moves closer towards the cavity. An alternating generation of hydrogen bond formation and water bridging of hydrogen bonds between the side chain of Arg127 and GlcN6P explains the movement of GlcN6P. The hydrogen bond network consisting of Ser37, Thr38, and Lys194 stabilizes GlcN6P within the active site. At this stage, the four residues described above are stable around the substrate, no new hydrogen bonds appearing. This implied the substrate is close to the active site and gradually became stabilized.

In the fifth stage (15.0Å ≥ RC ≥ 12.0 Å), GlcN6P has completely entered the active pocket, the residues having stabilized. The hydrogen bond network, is represented by the key residues Thr38, Ser37, Arg127, Lys194, and His130. Newly hydrogen bonds form between GlcN6P in the catalytically active center of the enzyme. At this point, no flipping is detected for the substrate mainly due to the strengthening of the stable hydrogen bond network.

**FIGURE 10:**
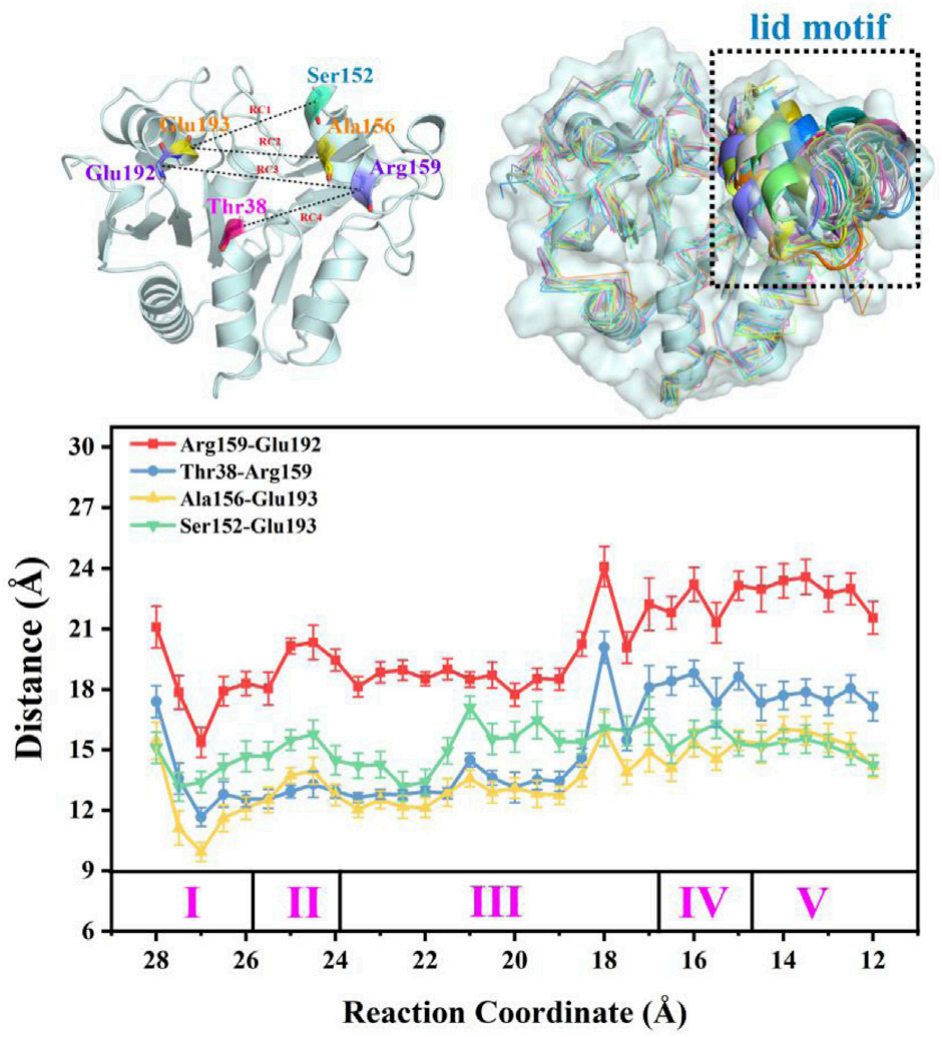
Overlap of the enzyme-substrate complex in alkaline solution at different stages and distances between key residues that reflect conformation changes to the lid motif during the substrate delivery process.

It is noteworthy that the performance of the “lid motif” is associated with substrate binding. In a previous study, models of apo-*Smu*NagB and the *Smu*NagB-GlcN6P complex in acidic and basic environments were established to compare conformational changes of the “lid motif” for preliminary forecasting. One trajectory for each model obtained by classic molecular dynamic simulation predicted different performance of both the “lid motif” and GlcN6P binding in acidic and basic environments. Regarding the latter, basic environmental conditions are required for subsequent enzymatic chemical reactions to occur. The “lid motif” has been shown to be open the apo and complexed state. Here, the detailed recognition and delivery process for GlcN6P in *Smu*NagB was analyzed. The conformation of the “lid motif” at pH = 8 was analyzed dynamically for greater reliability of results through the use of 33 windows in umbrella sampling simulations. Key distances for D1 (Ser152-Glu193), D2 (Ala156-Glu193), D3 (Arg159-Glu192), and D4 (Arg159-Thr38) reflected conformational changes to the “lid motif”, selected for measurement along the reaction coordinate of GlcN6P delivery, respectively. Thirty-three snapshots during the final 10 ns were selected for each window, the results of which are presented in Figure 10. Moreover, snapshots in key windows overlapped so that conformational changes could be ascertained more clearly. It can be observed in the first two stages that the “lid motif” region was open with slight fluctuations. During the third stage, the distance to the pocket clearly increased during the latter period, implying that opening of the “lid motif” become larger to allow substrate ingress. Subsequently, the lid motif started to close in comparison with the point at which the opening was greatest. During the fourth and fifth stages, the “lid motif” was more stable. It was open to a greater extent than during the first two stages but smaller than at the point located at RC = 18 Å, indicating that GlcN6P had already entered the “door” associated with the “lid motif”, and was therefore ready to dock with the site appropriate for the catalytic reaction. Our results agree well with previous predictions. Even more noteworthy is the considerably clearer picture of dynamic changes to the “lid motif” in the present study as GlcN6P entered the active site. Furthermore, changes in the interactions between key residues located in the “lid motif” and substrate reflect the critical points during substrate delivery. For example, changes in interactions between Arg159 and GlcN6P represent the status of GlcN6P. The presence of such an interaction demonstrates that GlcN6P has begun to enter the “door”, while its strengthening or its absence indicate that GlcN6P is close to the entrance of the pocket. The RMSF of NagB for each window is acquired that also implies the lid motif experiences the conformation changes, as listed in Supplementary Figure S9. The snapshots in the window with strongest fluctuation for “lid motif” in each stage are corresponding to the obvious opening behavior, which agree with each other well. Therefore, the behavior of the “lid motif” studied here is crucial for understanding the process by which the substrate is recognized and binds.

Analysis of the free energy distribution demonstrates that delivery of GlcN6P from the exterior to the active site is an exothermic process, releasing 8.27 kcal/mol, as displayed in Figure 9, representing the energy lost in subsequent chemical reaction, to some extent. During the first stage, which results in a steady loss of 0.36 kcal/mol, GlcN6P is recognized primarily by the key residues Arg159 and Lys9. During the second stage, a rapid decrease of 3.06 kcal/mol is observed, principally caused by the flipping of the carbohydrate ring from a vertical to a horizontal position and the formation of new hydrogen bonds between GlcN6P and residues Lys194 and Glu193. During the third stage an exothermic process that releases 4.85 kcal/mol, is characterized most by the growing number of hydrogen bonds which provide sufficient traction for the substrate to move further. It should be noted that within the region 21.5Å > RC > 18.5 Å, recently formed hydrogen bonds between the substrate and Thr38 and Ser37 play a dominant role in guiding energy release. At RC = 18.5 Å, the energy of the system is at its minimum point. Here, the “lid motif” is at its greatest opening. At 18.5Å > RC > 17.5 Å, the energy balance displayed a weak uptrend possibly caused by the breaking of water bridged hydrogen bonds and the slight closing of the “lid motif”. During the fourth stage, free energy firstly decreased and then increased. The decrease in energy probably arises from changes in hydrogen bonding between the substrate and Thr38, Ser37, Arg159, and Lys194. Direct hydrogen bonding replaced indirect water bridged hydrogen bonds, the strength of which increases. Subsequent increases in energy may arise from decreased direct hydrogen bond interactions between these four residues and the substrate. Furthermore, the flipping of GlcN6P probably also resulted in a rise in energy. During the fifth stage, free energy displayed a decreasing trend of 2.10 kcal/mol, indicating that the substrate become gradually located in a more energetically favorable location with a greater number of interactions. Fresh hydrogen bonds from residues His130 and Gly132 provide the key driving force for GlcN6P binding and stabilization. As described above, changes in free energy mainly arise from changes in both direct and indirect hydrogen bond interactions. The flipping of GlcN6P and conformational changes to the “lid motif” also provided a small contribution. Here, the participation of Arg159, Lys194, Thr38, Ser37, His130, and Gly132 should be noted, since the new hydrogen bonds formed with the substrate are crucial in driving movement of the substrate towards the active site of the enzyme. They also allow a release of energy from the substrate at the entrance the subsequent chemical reaction.

## Conclusion

**FIGURE 11:**
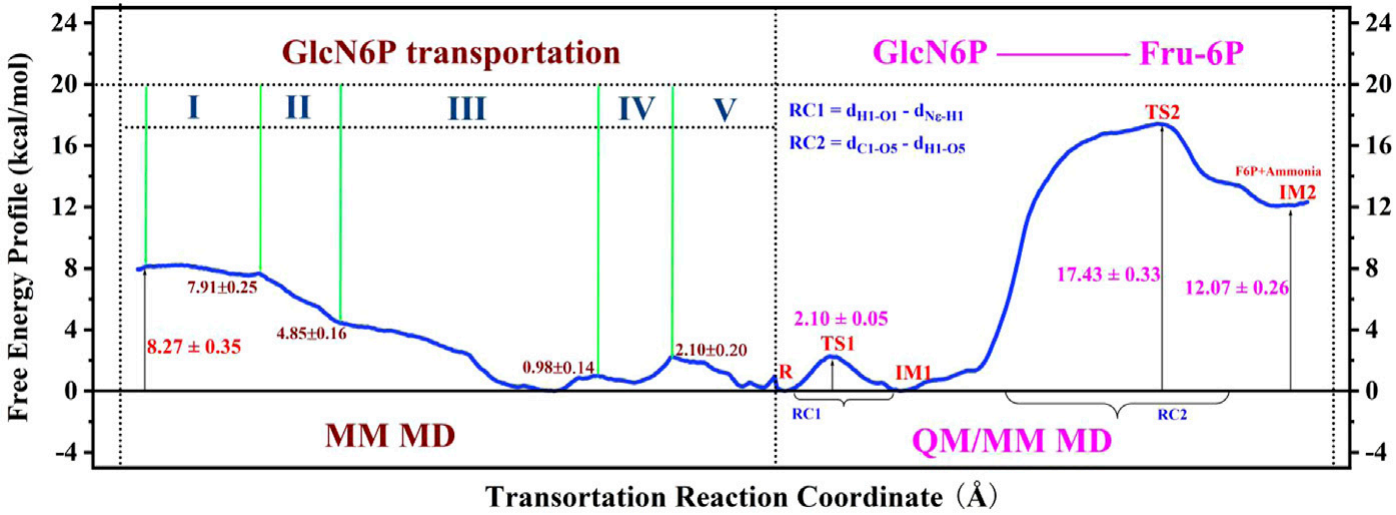
Entire free-energy profile for GlcN6P transportation, and protonation, according to MM MD and QM/MM MD simulations.

In the present work, MM MD simulations combined with MM/GBSA, RAMD MD, and umbrella sampling were performed to study the processes of recognition and delivery for GlcN6P by *Smu*NagB. Four novel aspects to these processes were observed. Firstly, the hydrogen bond network formed between GlcN6P and Ser37, Thr38, His130, Gly132, and Lys194 plays a crucial role in stabilizing GlcN6P in the active site, the electrostatic and polar interactions of which predominant compared with van der Waals and non-polar interactions. The energy decomposition of the four mutant models, namely Ser37, Thr38, His130, and Lys194, confirm these observations. Secondly, four channels for GlcN6P delivery were observed, and the “door” of the P1 channel located on residues 6–10, 122–136, and 222–233 accounted for 51.79% of the most favorable access channels. Movement of GlcN6P to the active site along P1 is an exothermic process, releasing 8.27 kcal/mol of energy and highly dependent on direct and indirect water bridging hydrogen bond interactions. The residues Arg159, Lys194, Thr38, Ser37, His130, and Gly132 provide the principal driving force for substrate delivery from outside of the active site of *Smu*NagB. Flipping of GlcN6P and conformational variations in the “lid motif” are also responsible for changes in free energy. Thirdly, the “lid motif” in *Smu*NagB is greatly associated with substrate recognition and the binding process. It is always “open” within a basic solution (pH = 8), allowing the substrate to cross the threshold of the protein. Variations in this motif represent different stage of GlcN6P delivery, especially the third stage, where it displays the greatest opening. It closes to a small extent during the last two stages due to the pocket being larger than during the first two stages, clearly associated with movement of the substrate. Finally, access of the reactant and the ring-opening step in the chemical reaction (Zhao et al., 2014) is displayed in Figure 11 revealing that for the enzymatic process, reactant binding provides the initial driving force for the subsequent chemical reaction to occur due to release of energy. Nevertheless, the chemical reaction requires additional energy and so additional driving forces probably exist, possibly from isomerization and deamination processes, in addition to the release of the products, F6P and ammonia. The present work provides the theoretical basis from an atomic standpoint that elucidates the enzymatic catalysis by NagB. Furthermore, designs of inhibitors with superior dynamic behavior that target *Smu*NagB in *S. mutant*, is a major caries-causing bacterium in humans and animals, may utilize these insights, and so we anticipate that the results will accelerate associated research progress, both experimentally and theoretically.

## Data Availability

The original contributions presented in the study are included in the article/Supplementary Material, further inquiries can be directed to the corresponding authors.
