# Supplementary material for: Atomic-Level Investigation of Reactant Recognition Mechanism and Thermodynamic Property in Glucosamine 6-Phosphate Deaminase Catalysis
Source: Front Chem. 2021 Aug 3;9:737492. doi: 10.3389/fchem.2021.737492 (PMC8369159; doi:10.3389/fchem.2021.737492)
Supplement: Supplementary file 1 [file DataSheet1.DOCX]

Supplementary Material

Atomic-level investigation of reactant recognition mechanism and thermodynamic property in glucosamine 6-phosphate deaminase catalysis

Xiao Zhang^1^, Xiaoyuan Liu^1^, Zhiyang Zhang^1^, Yuan Zhao^1*^, Chaojie Wang^1*^

[a] The Key Laboratory of Natural Medicine and Immuno-Engineering, Henan University, Kaifeng 475004, People’s Republic of China

*Corresponding Authors

E-mail addresses: zhaoyuan@henu.edu.cn (Y.Zhao), wcjsxq@henu.edu.cn (C.Wang).


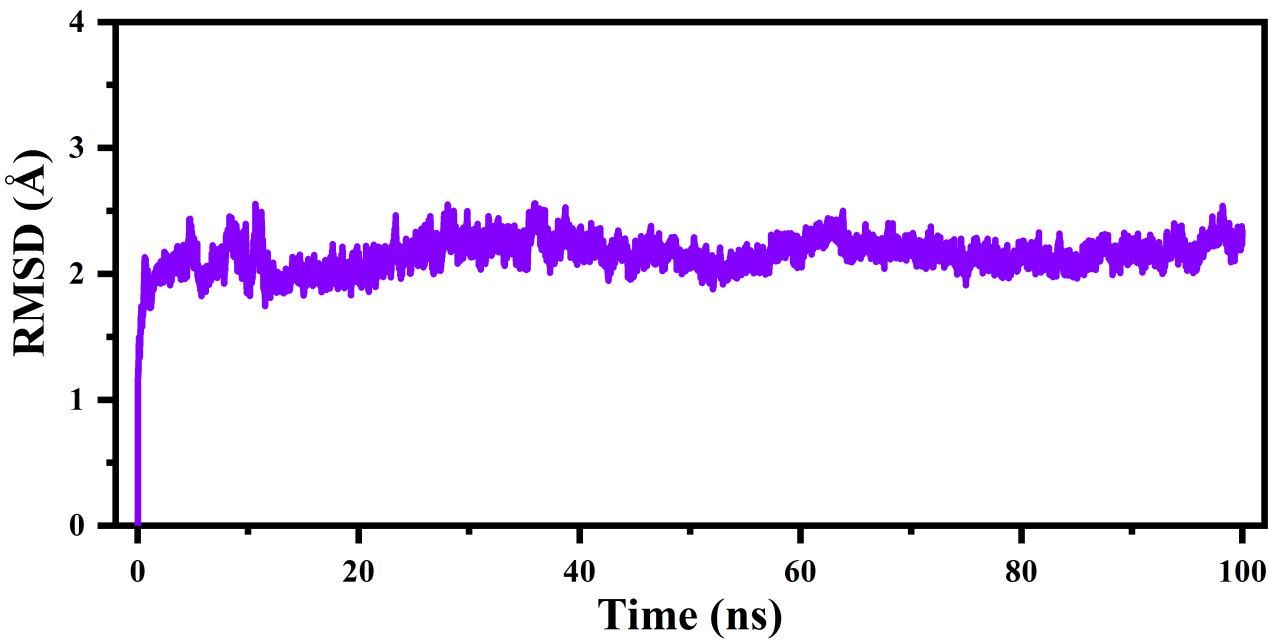


**Supplementary Figure 1.** The root mean square deviation (RMSD) of all the backbone atoms by 100 ns MM MD simulations.

**(a)**


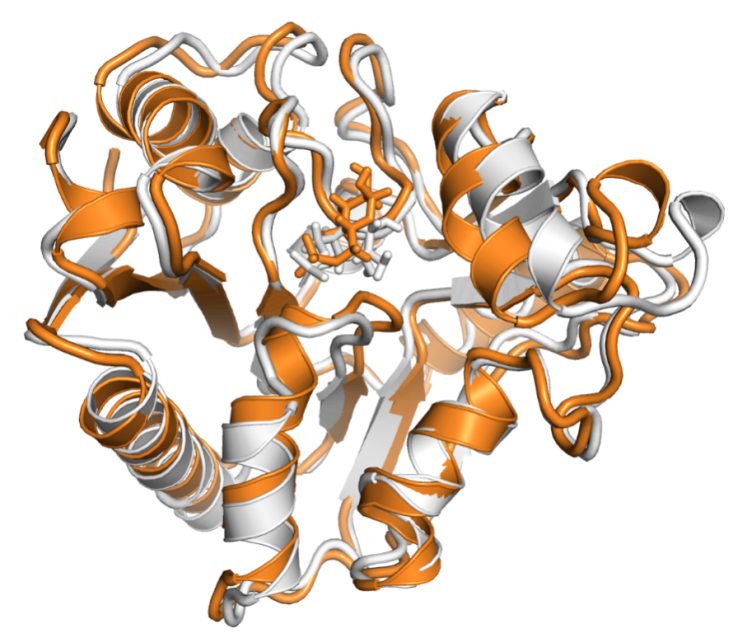


**(b)**


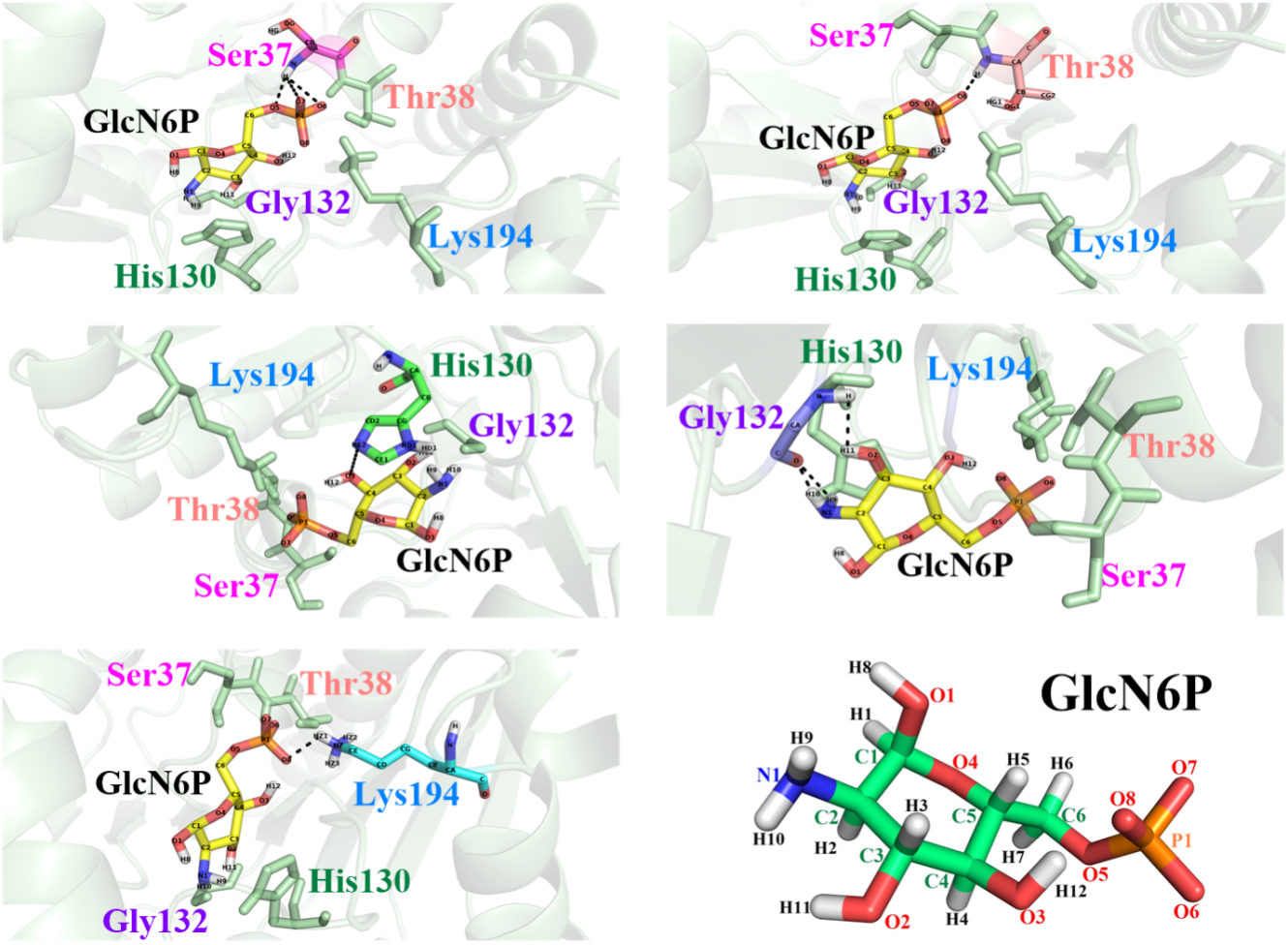


**Supplementary Figure 2.** **(a)** Overlap of snapshots after equilibrium in MM MD simulations (colored in orange) and crystal structure in experiment (colored in white). **(b)** The number of key atoms in hydrogen bond analysis.


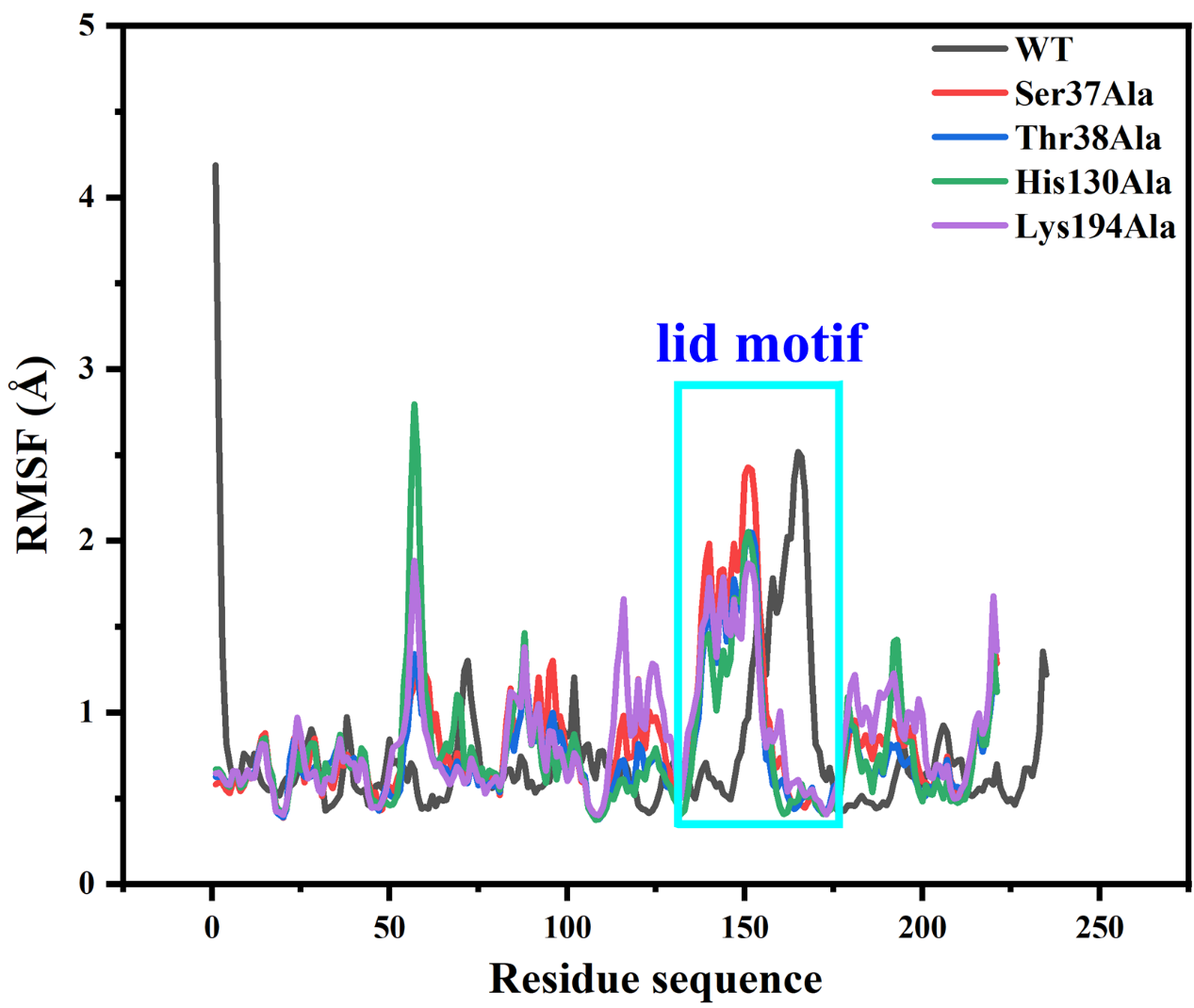


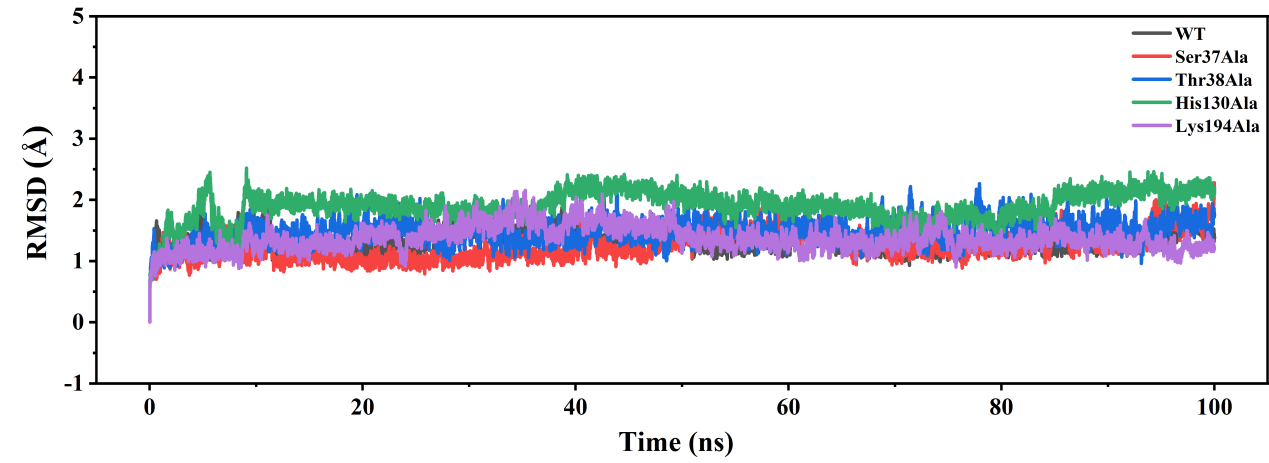


**Supplementary Figure 3.** The RMSD and RMSF values of all the backbone atoms as function of time for the SmuNagB-GlcN6P complex in alkaline solution.


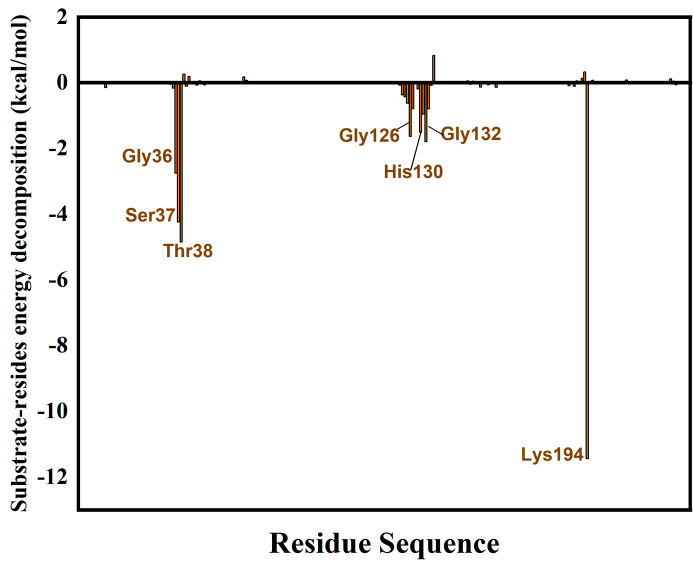


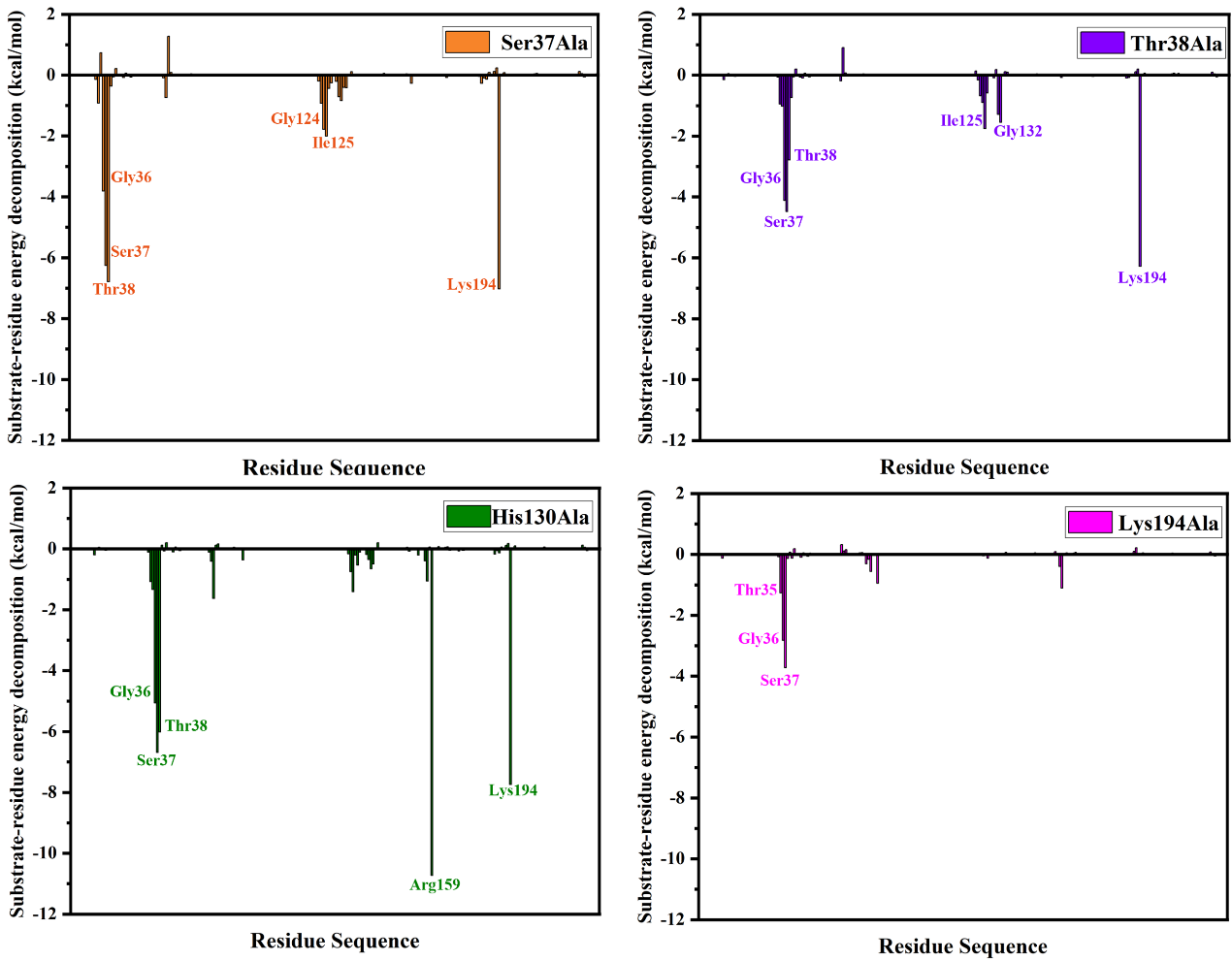


**Supplementary Figure 4.** The RMSD and RMSF values of all the backbone atoms as function of time for the SmuNagB-GlcN6P complex in alkaline solution.


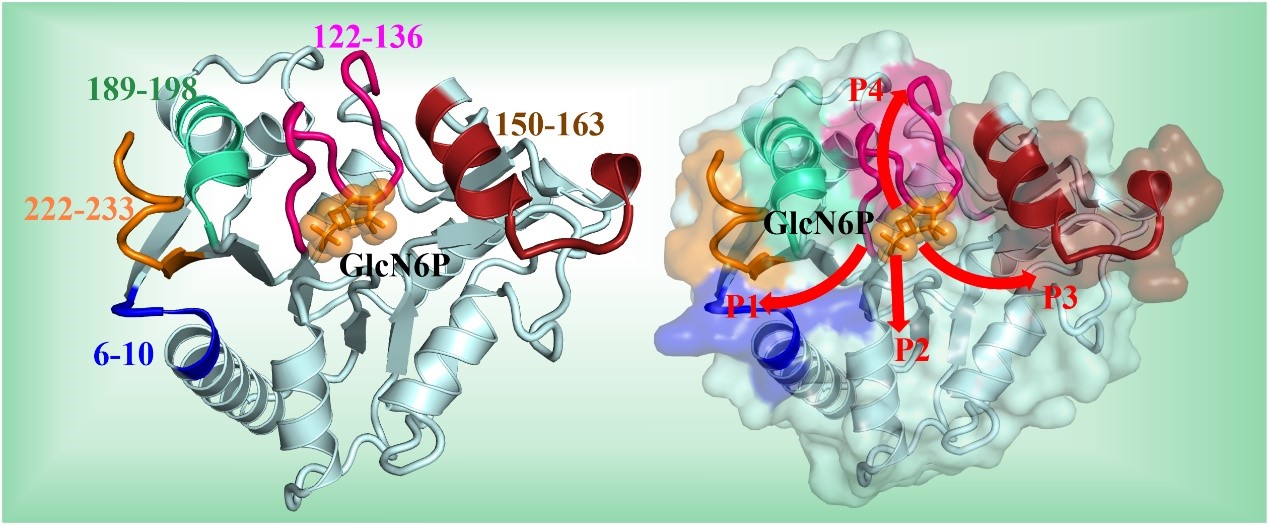


**Supplementary Figure 5.** Distributions of residues involved in the possible pathways P1, P2, P3, and P4, for the reactant recognize based on the RAMD MD simulations.


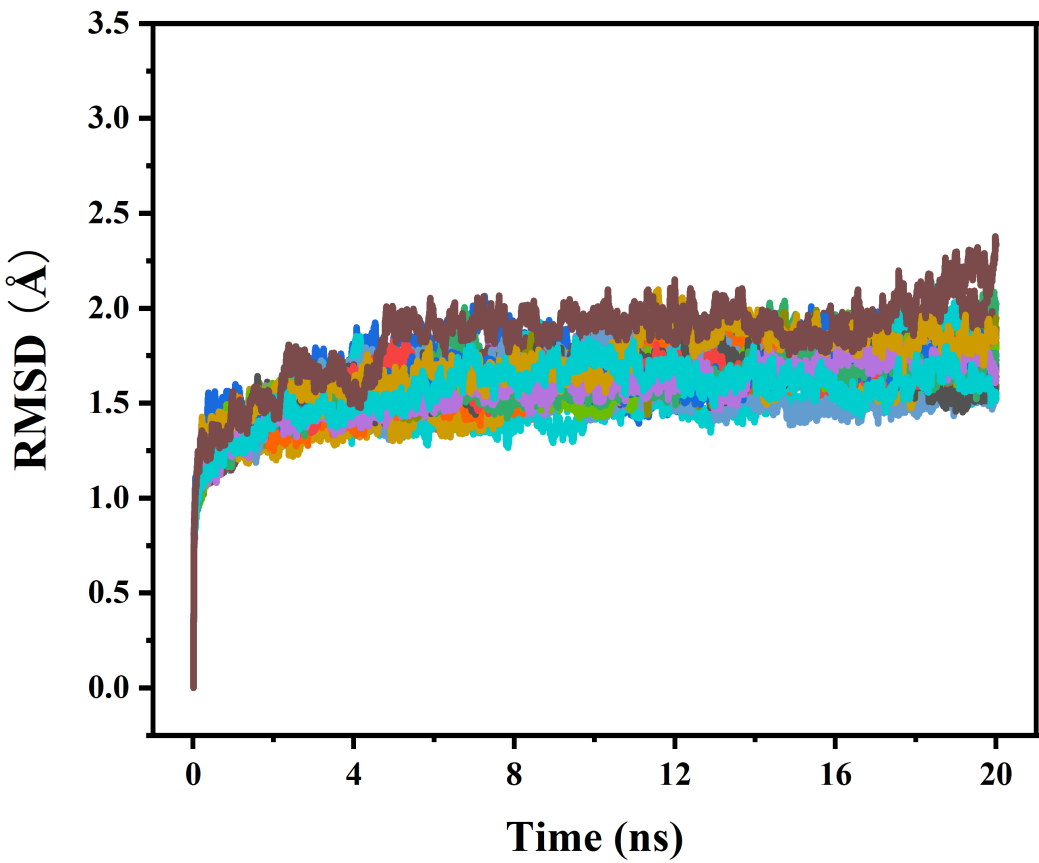


**Supplementary Figure 6.** The root mean square deviation (RMSD) of all the backbone atoms by total 660 ns classical MD simulations combined with umbrella sampling technique along P1 pathway.


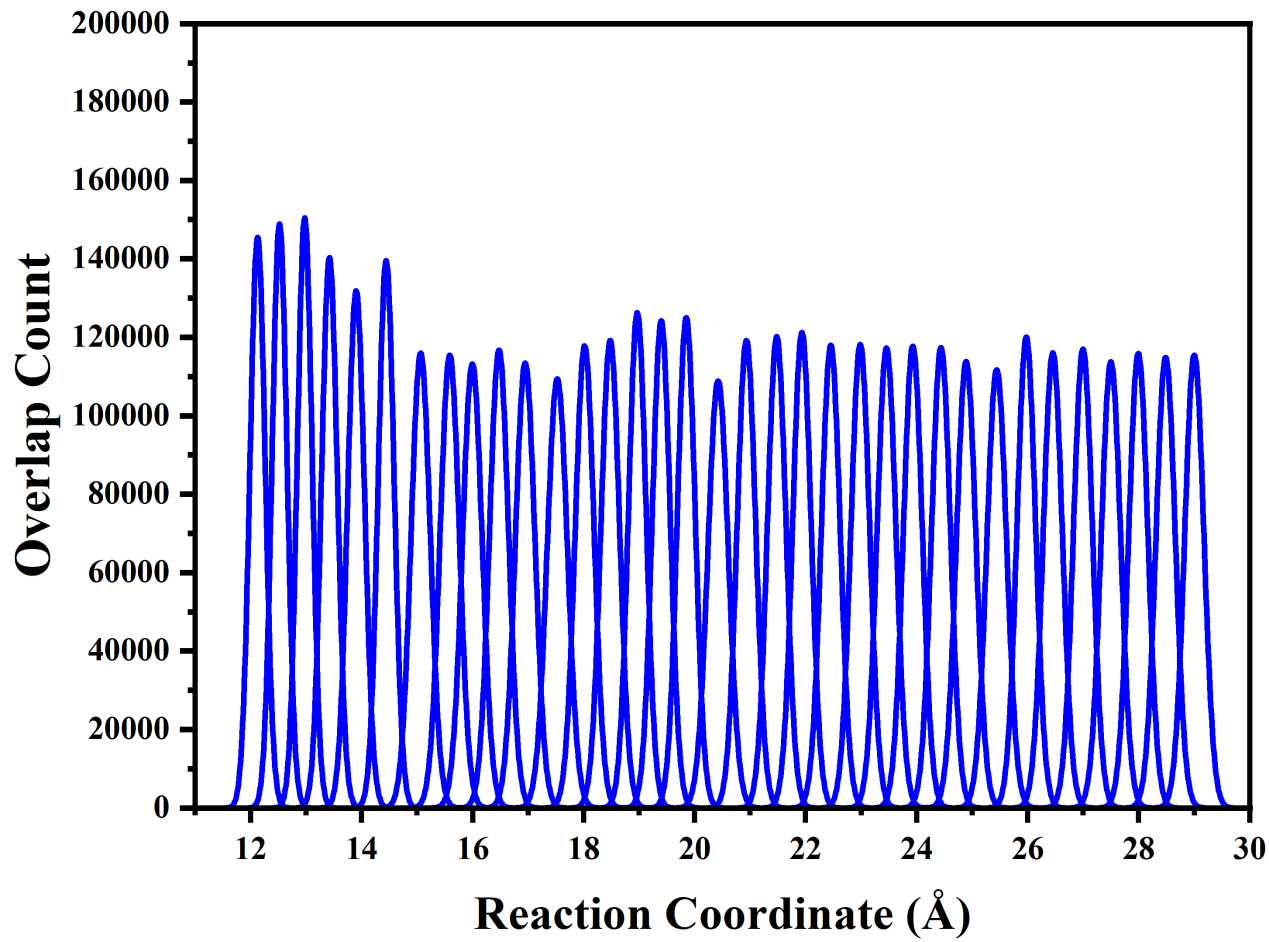


**Supplementary Figure 7.** The overlap of windows along P1 pathway according to umbrella sampling technique.


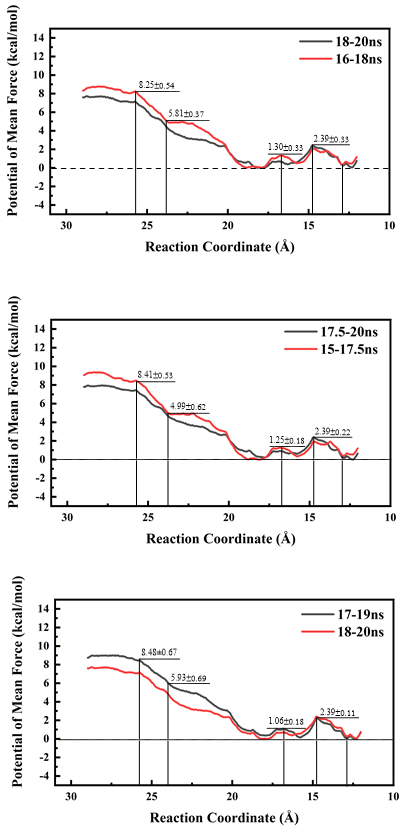


**Supplementary Figure 8.** Potential mean force (PMF) of GlcN6P recognition and delivery along the P1 channel evaluated by MM MD simulations incorporating an umbrella sampling technique for different time periods.


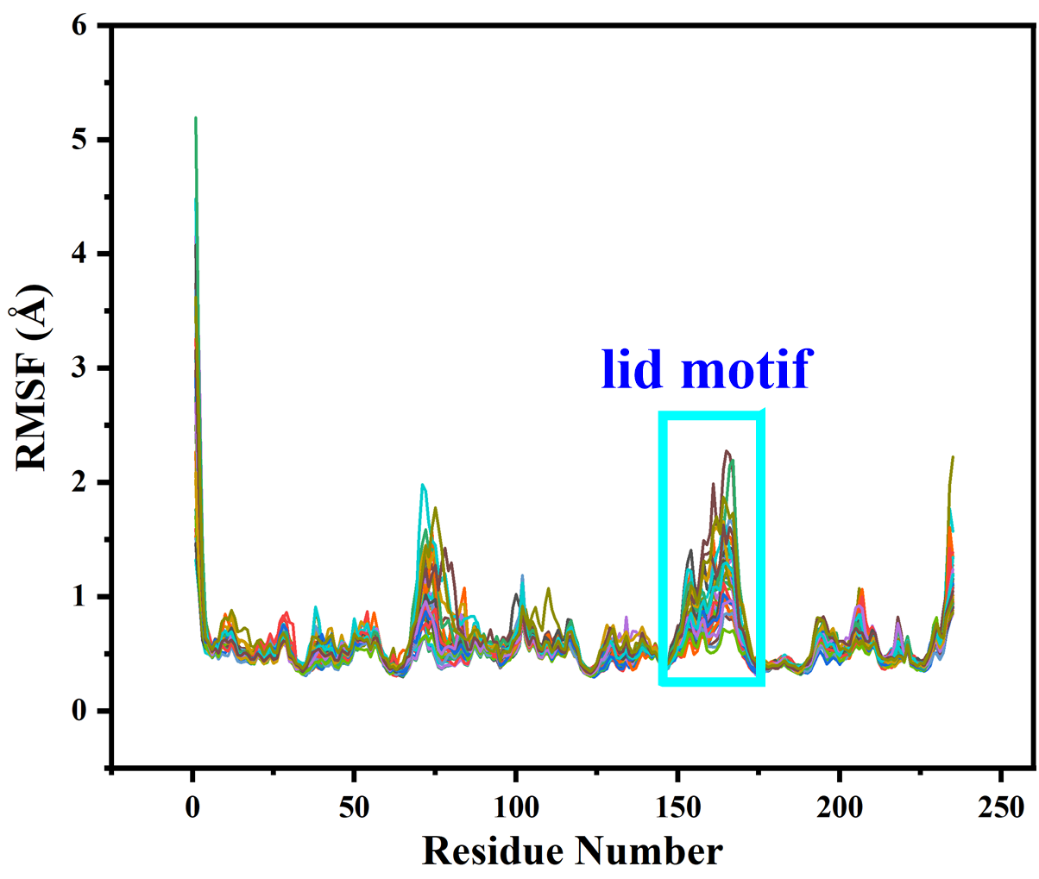


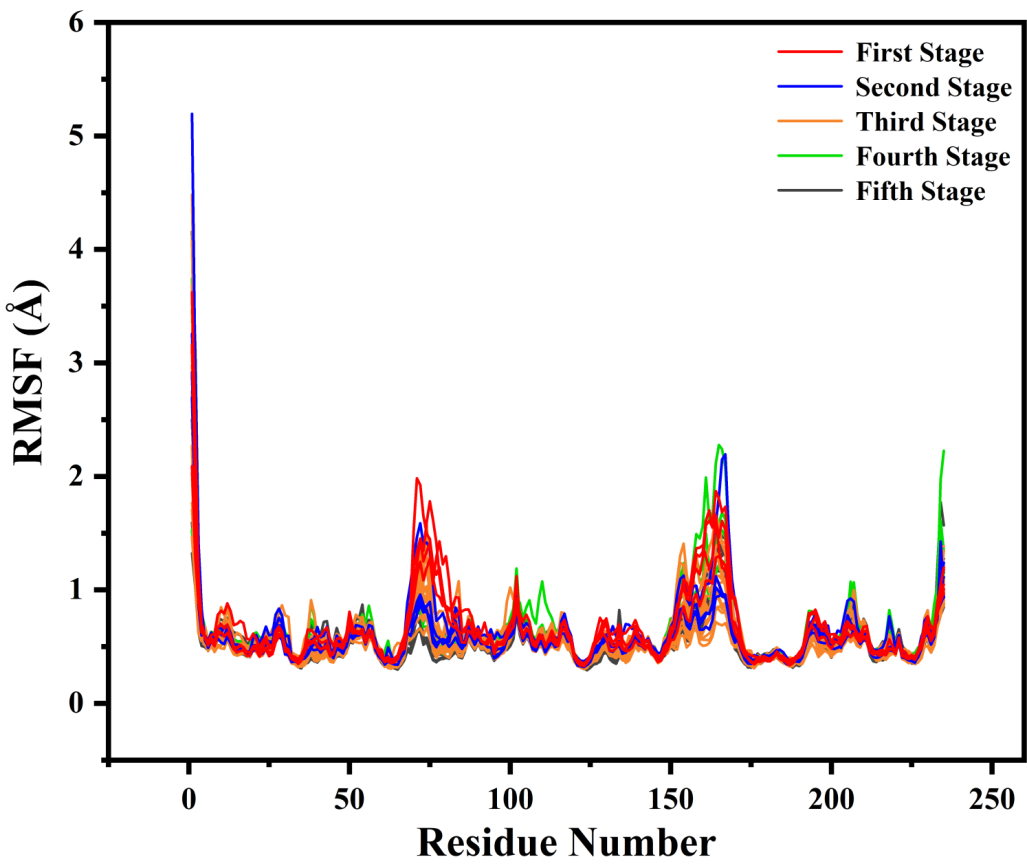


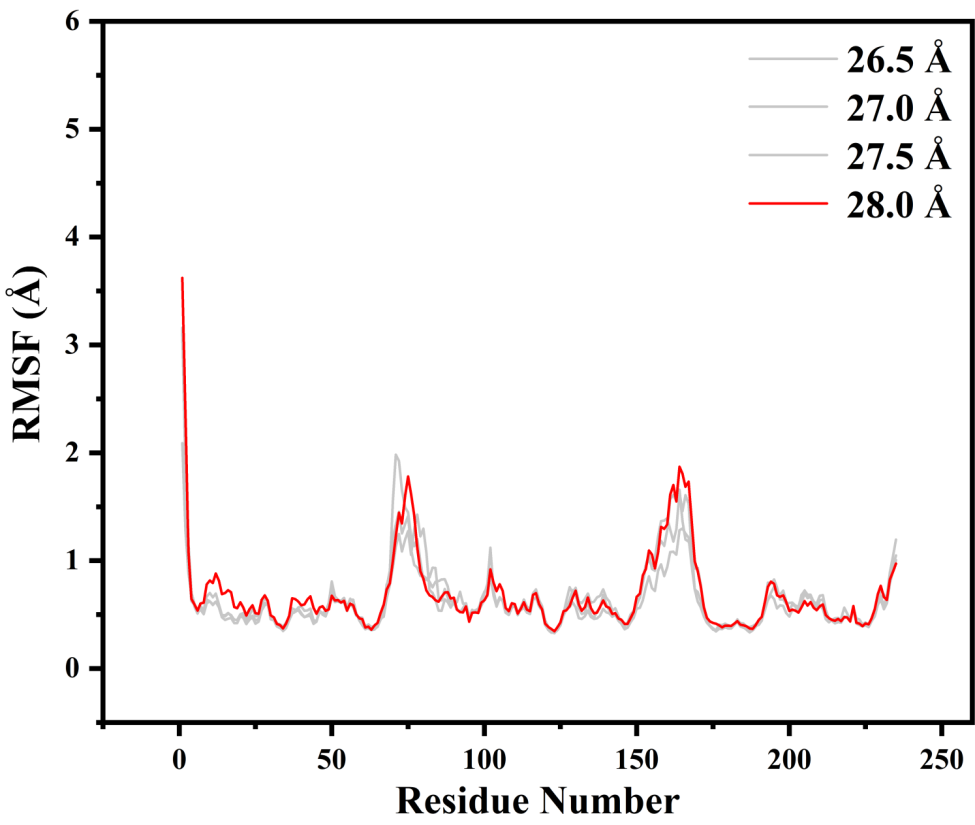


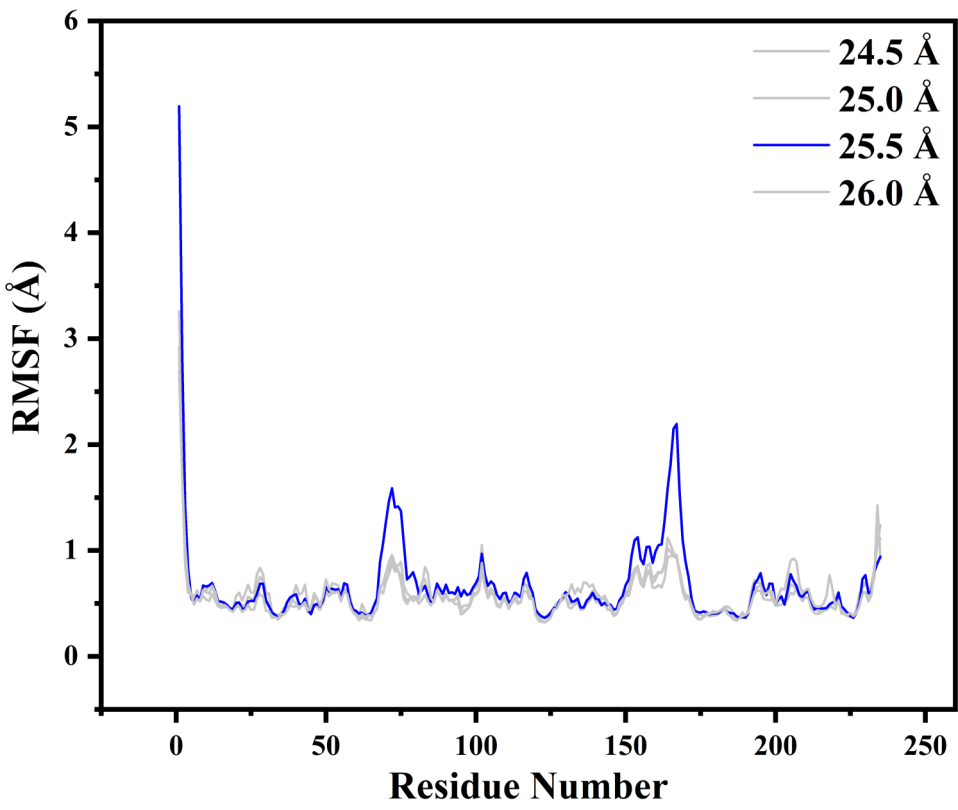


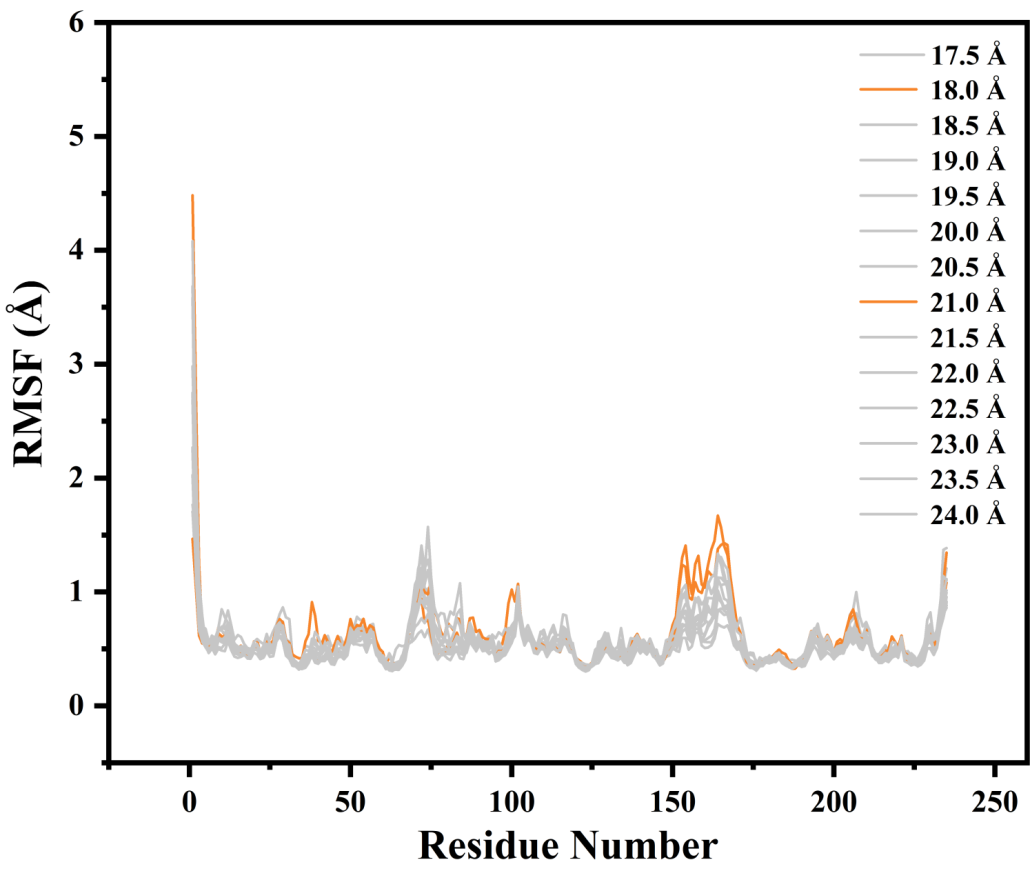


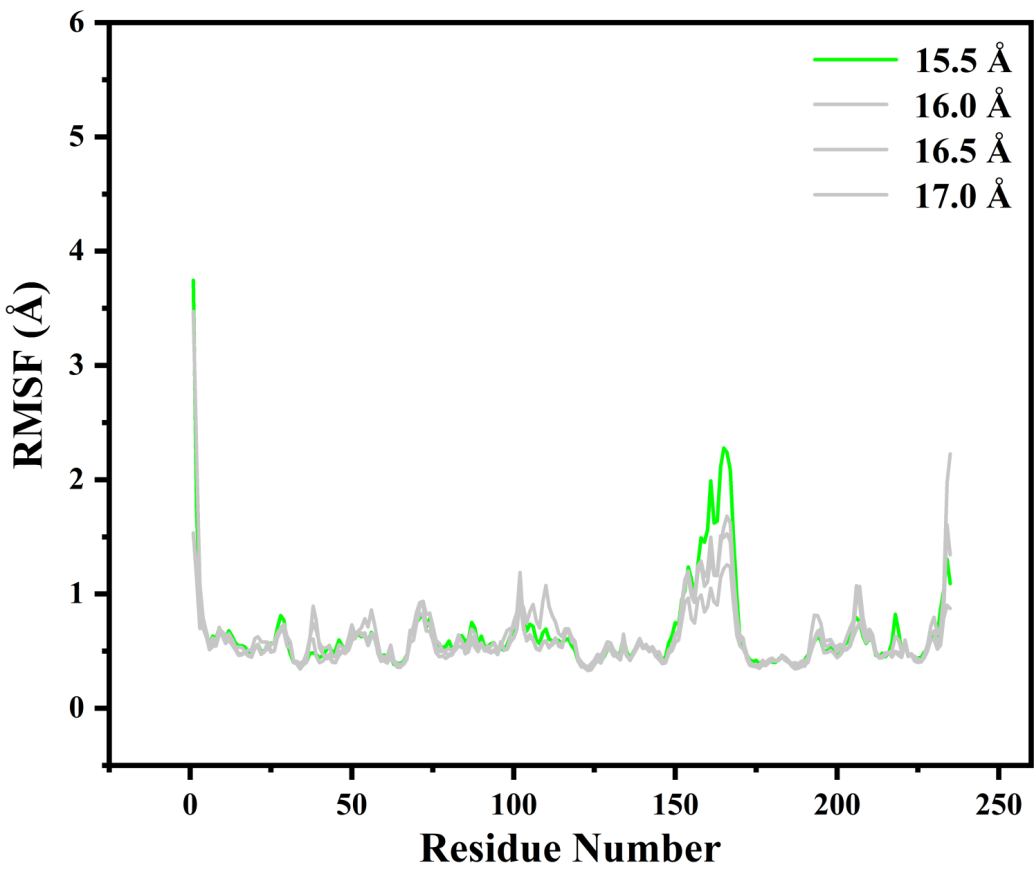


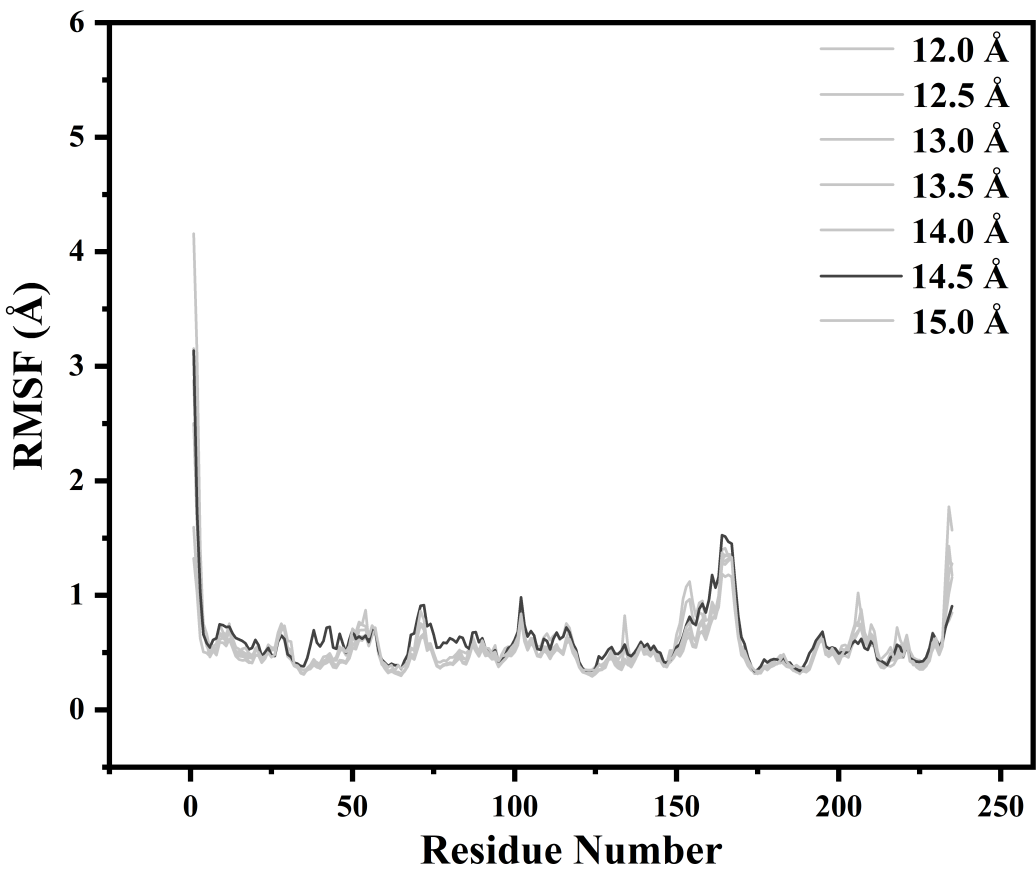


**Supplementary Figure 9.** The RMSF of NagB for each window obtained by 33 snapshots in MM MD simulations combined with umbrella sampling technique. The I-V stages are colored with red, blue, orange, green, and black.
